# Antioxidant and Antiglycation Effects of *Cistus × incanus* Water Infusion, Its Phenolic Components, and Respective Metabolites

**DOI:** 10.3390/molecules27082432

**Published:** 2022-04-09

**Authors:** Karolina Bernacka, Katarzyna Bednarska, Aneta Starzec, Sylwester Mazurek, Izabela Fecka

**Affiliations:** 1 Department of Pharmacognosy and Herbal Medicines, Faculty of Pharmacy, Wroclaw Medical University, ul. Borowska 211, 50-556 Wrocław, Poland; katarzyna.bednarska@student.umed.wroc.pl (K.B.); aneta.starzec@student.umed.wroc.pl (A.S.); izabela.fecka@umw.edu.pl (I.F.); 2 Laboratory of Chemometrics and Applied Spectroscopy, Department of Chemistry, University of Wrocław, 14 F. Joliot-Curie, 50-383 Wrocław, Poland; sylwester.mazurek@chem.uni.wroc.pl

**Keywords:** *Cistus × incanus*, polyphenols, flavonols, urolithins, antioxidants, glycation inhibitors, methylglyoxal trapping, PCA, PLS-DA

## Abstract

Reactive oxygen and carbonyl species promote oxidative and carbonyl stress, and the development of diabetes, metabolic syndrome, cardiovascular diseases, and others. The traditional herb *Cistus × incanus* is known for its antioxidant properties; therefore, the current study aimed to assess how the chemical composition of a *C. incanus* water infusion corresponds with its antioxidative and antiglycative effects in vitro. The composition of infusions prepared from commercial products was analyzed with UHPLC-ESI-qTOF-MS. Total phenolics, flavonoids, and non-flavonoid polyphenols were determined. Antioxidant activity of infusions and selected polyphenols was investigated using DPPH, ABTS, and FRAP. Fluorometric measurements and methylglyoxal capture were performed to investigate the antiglycation activity. PCA and PLS-DA models were applied to explore the correlation between chemical and antioxidant results. The principal flavonoids in *C. incanus* were flavonols. In vitro tests revealed that a stronger antioxidant effect was demonstrated by plant material from Turkey rich in flavonoids, followed by Albania and Greece. Flavonols and ellagic acid displayed stronger antiradical and reducing power than EA-derived urolithins. Hyperoside was the most potent inhibitor of glycation. The results indicate that flavonoids are primarily responsible for rock rose antioxidant and antiglycation properties. PLS-DA modeling can be used to identify the origin of plant material with sensitivity and specificity exceeding 86%.

## 1. Introduction

*Cistus × incanus* L., commonly known as hairy or pink rock rose, is considered to be a great source of antioxidants for the food and pharmaceutical industry [1]. Since 2010 the European Food Safety Authority (EFSA) has recommended *C. incanus* water infusions as a natural source of antioxidants, which improve the antioxidant status of the organism and support the immune system [2]. An imbalance between endogenous antioxidants and pro-oxidants is associated with the occurrence of oxidative stress [3]. The deteriorating effect of oxidative stress underlines the aging process and the development of non-communicable chronic diseases [4,5]. Numerous studies have confirmed that oxidative stress plays an essential role in the pathogenesis of diabetes mellitus, its vascular complications, cardiovascular diseases, kidney disease, and neurodegenerative disorders such as Alzheimer’s disease [6,7]. Besides ROS (reactive oxygen species), involved in inducing oxidative stress, AGEs (advanced glycation end products) and their precursors—reactive carbonyl species (RCS, e.g., dicarbonyls)—also act as prooxidants and proinflammatory agents [8]. Both AGEs and RCS, such as methylglyoxal and glyoxal, underline the pathogenesis of chronic conditions including dyslipidemia, non-alcoholic fatty liver, atherosclerosis, obesity, metabolic syndrome, insulin resistance, prediabetes, diabetes, and cancer [8,9,10].

The object of our investigation, *Cistus × incanus* L., is a perennial, herbaceous plant endemic to the Mediterranean area [11,12]. The taxonomy of *Cistus* sp. is complex, which may cause problems with the identification of particular species. Plants in this genus tend to cross between species and form hybrids [13]. *C. incanus* is a hybrid of two species: *C. albidus* and *C. crispus* [14]. Within the genus *C. incanus*, three subtypes are recognized: *C. incanus* subsp. *incanus*, *C. incanus* subsp. *Corsicus*, and *C. incanus* subsp. *creticus* (also known as *C. creticus* or *C. villosus*) [13,15]. *Cistus* subspecies vary in leaf morphology, shape, and the content of active compounds [15,16]. They are distinguished by the presence of glandular trichomes that secrete a resin called labdanum (or ladano). The resinous exudate from rock rose is a source of terpenoids such as mono-, di-, sesquiterpenes, and labdane-type diterpenes [11,15]. To alleviate the adverse effect of oxidative stress, which occurs as a consequence of excessive sun irradiation in the Mediterranean environment, plants produce secondary metabolites, for instance, polyphenols [17]. Flavonoids and tannins are major groups of phenolic compounds, occurring in *C. incanus* [12,18]. Among the flavonoids identified in extracts, there are flavonol derivatives, e.g., myricetin, quercetin, kaempferol, and their glycosides, including myricitrin, quercitrin, hyperoside, and tiliroside [12,18,19,20,21]. Non-flavonoid polyphenols present in *C. incanus* include tannins such as proanthocyanidins (also called condensed tannins), and hydrolyzable tannins, mostly esters of glucose with hexahydroxydiphenic acid [18,22]. Ellagitannins such as punicalin, punicalagin, terflavin A, and cistusin are significant constituents found in the *C. incanus* water infusion and other extracts [18,23]. Phenolic acids are a well-described small group of phenolics which include mainly gallic acid and smaller quantities of protocatechuic, *p*-coumaric, chlorogenic, and ellagic acids [19,24].

The specific chemical composition of *C. incanus* is responsible for the multidirectional properties of this plant. Traditional medicine used *Cistus* species for anti-inflammatory, antimicrobial, antispasmodic, antiulcerogenic, wound healing, and vasodilator curatives [12,18]. It is also worth noting that *C. creticus* derived from local floristic resources has been used as an antidiabetic agent in Morocco [25]. Recently, numerous studies have confirmed the validity of applying rock rose in traditional medicine. Apart from its antioxidant properties, this raw plant material exhibits anti-inflammatory [26], antibacterial [18,27,28], antiviral [29,30], antifungal [31], antiproliferative, and cytotoxic activity [32]. In recent work, the influence of *C. incanus* on COVID-19 has been investigated as well [33]. Last year, Kuchta et al. [24] demonstrated that systematic administration of *C. incanus* water infusion decreased cardiovascular risk factors including oxidative stress and dyslipidemia by improving the lipid profile, increasing HDL-C concentration, and reducing triacylglycerol, serum malondialdehyde, and advanced oxidation protein product (AOPP) levels. 

Although the chemical composition of the rock rose has been widely described, the specific group of biologically active compounds providing its antioxidant properties has not been formally defined [1]. Thus, our study aimed to investigate the relationship between phenolics, flavonoids, non-flavonoid polyphenols, and the antioxidant activity of *C. incanus* leaves from different countries. To complement the study, we examined the antioxidant effects of selected components of *C. incanus*, such as flavonol glycosides and aglycones, and ellagitannin metabolites, including ellagic acid and urolithins. Since flavonoids are known for their ability to inhibit AGE formation and AGE-induced inflammation [26,34], we also investigated the antiglycation activity of *C. incanus* infusions, their major chemical constituents, and potential gut microbiota metabolites. Next, to better understand the mechanism of antiglycation activity, we evaluated the ability to form methylglyoxal adducts, both for infusions and rock rose components selected by UHPLC-ESI-qTOF-MS. Unfortunately, many commercial *C. incanus* products do not have information on the country of origin of the plant material; therefore with the help of chemometrics and spectrophotometric results, we developed a simple protocol for their tentative identification.

## 2. Materials and Methods

### 2.1. Chemicals and Apparatus

Folin-Ciocalteu reagent, sodium bicarbonate (Na_2_CO_3_), iron(III) chloride (FeCl_3_), and methanol were obtained from Chempur (Poland). Aluminium(III) chloride (AlCl_3_) and 2,4,6-Tris(2-pyridyl)-s-triazine (TPTZ) were purchased from Fluka (Buchs, Switzerland); DPPH, ABTS, methylglyoxal (40% in water), methanol (HPLC grade), acetonitrile (HPLC gradient grade and LC-MS grade), water (LC-MS grade), bovine serum albumin (BSA), DMSO, and 98–100% formic acid from Sigma-Aldrich (St. Louis, MO, USA); iron(II) sulfate heptahydrate (FeSO_4_ · 7 H_2_O), NaCl, KCl, Na_2_HPO_4_, KH_2_PO_4_ (reagent grade), and others from Chempur (Piekary Śląskie, Poland).

All spectrophotometric measurements were conducted using a Multiskan GO Spectrophotometer (Thermo Fisher Scientific, Waltham, MA, USA).

### 2.2. Plant Material

The plant material used in this study consisted of 51 commercially available products containing dried *Cistus* × *incanus* L. leaves or herb (Ci1–Ci51). The products were purchased in Poland and were obtained from different manufacturers. Raw plant material came from different countries—Turkey, Albania and Greece—or had an unknown origin.

### 2.3. Preparation of Water Infusions

Infusions were prepared in duplicate. First, freshly boiled distilled water (200 mL) was added to dried plant material (1.5 g). The infusions were brewed under cover and after 15 min filtered through cotton wool, and next through a membrane filter with pore size 0.45 μm (Durapore; Millipore, Burlington, MA, USA).

### 2.4. Reference Standards

The following reference standards were used: gallic acid (CAS No.: 149-91-7) was purchased from Extrasynthese (Genay, France); myricetin (CAS No.: 529-44-2) from Fluka AG (Switzerland); kaempferol (CAS No.: 520-18-3), quercetin (CAS No.: 117-39-5), hyperoside (CAS No.: 482-36-0), myricitrin (CAS No.: 17912-87-7), ellagic acid (CAS No.: 476-66-4), Trolox (CAS No.: 53188-07-1), aminoguanidine hydrochloride (CAS No.: 79-17-4) and metformin hydrochloride (Pharmaceutical Secondary Standard) (CAS No.: 657-24-9) from Sigma-Aldrich. Urolithin A, urolithin B, urolithin C were synthesized according to Bialonska et al. [35]. The purity of all urolithins was ≥95% (determined by HPLC-PDA, column: Hypersil GOLD, 5 µm, 250 × 4.6 mm; Thermo Fisher Scientific; Waltham, MA, USA). 

To determine the antioxidant and antiglycation effects of chemicals occurring in the *C. incanus* leaves, methanolic or DMSO solutions of reference compounds were prepared. The stock solutions at concentration 3 mM were prepared using methanol to dissolve gallic acid, kaempferol, quercetin, myricetin, hyperoside, urolithins A, B and C, and DMSO to dissolve myricitrin and ellagic acid. Obtained working solutions were afterward diluted by methanol to concentrations ranging from 5 to 500 µM, and filtered by Durapore 0.22 μm filters (Millipore). Aminoguanidine and metformin were dissolved in a water-methanol solution (1:1, *v*/*v*).

### 2.5. Total Phenolic Content (TPC)

The total phenolic content was determined by the Folin-Ciocalteu method described by Singleton et al. [36], with some modifications. Briefly, 200 μL of water infusion was mixed with 40 μL of Folin-Ciocalteu reagent in Eppendorf tubes, and 800 μL of 10% Na_2_CO_3_ (*m*/*v*) was added after 5 min. Samples were incubated at room temperature in darkness for 30 min and centrifuged for 7 min, at the speed of 15 100 revolutions per minute (MPV MED. INSTRUMENT, Centrifuge MPV-260; Poland). All samples were prepared in triplicate. Next, 50 μL of each sample was applied to a 96-well microplate. The absorbance was measured at a wavelength of 725 nm.

The calibration curve was prepared for gallic acid (at concentrations of 0.01–0.1 mg/mL). Results were expressed as mg of gallic acid equivalents per gram of dry weight (mg GAE/g d.w.).

### 2.6. Total Flavonoid Content (TFC)

The total flavonoid content was determined according to the European Pharmacopoeia method for *Betulae folium* [37], with some modifications. First, 50 μL of 2% AlCl_3_ (*m*/*v*) and 50 μL of water infusions were applied to a 96-well microplate and incubated at room temperature in darkness for 60 min. The absorbance was measured at a wavelength of 420 nm. All samples were prepared in triplicate.

The use of quercetin as a standard had been rejected in favor of myricetin, on the grounds that myricetin derivatives occur in *C. incanus* in a greater amount [24,27]. The calibration curve was prepared at concentrations of 0.005–2 mg/mL. The TFC was expressed as mg of myricetin equivalents per gram of dry weight (mg ME/g d.w.).

### 2.7. UHPLC-ESI-qTOF-MS Method

The chemical composition of *C. incanus* water infusions and their ability to form adducts with methylglyoxal were analyzed using a Thermo Scientific DionexUltiMate 3000 UHPLC system (Thermo Fisher Scientific; Waltham, MA, USA) coupled with a Compact ESI-qTOF mass spectrometer (Bruker Daltonics; Bremen, Germany), a standard quaternary pump (LPG-3400D), a UltiMate 3000 RS autosampler (WPS-3000), and a rapid separation photodiode array detector (DAD-3000 RS Rapid Separation Diode Array Detector). The separation of compounds was carried out on a Kinetex C18 column (150 × 2.1 mm × 2.6 μm) (Phenomenex; Torrance, CA, USA), and a thermostatted column compartment (TCC-3000) was used to maintain its temperature at 40 °C. The mobile phases comprised 0.1% formic acid in water (A) and 0.1% formic acid in 100% acetonitrile (B). The following mobile phase gradient program was used at a flow rate of 0.3 mL/min: 0–12 min, 97–65% A in B; 12–14 min, 65% A in B; 14–17 min, 65–20% A in B; 17–19 min 20% A in B. Afterwards, the system was returned to the starting setting and washed with 97% A in B until the system was stabilized before the next analysis. For data acquisition, both positive and negative ion modes were applied. The injection volume was 2.5 μL. Nitrogen at 210 °C, 2.0 bar pressure, and 0.8 L/min flow rate was used as the nebulizing and drying gas. For internal calibration, sodium formate clusters at 10 mM were used. The capillary voltage was set to 5 kV (ESI+, ESI−), the collision energy was 8.0 eV, and the MS/MS mode was 35 and 40 eV. Data were acquired and analyzed using Compass Data Analysis software (Bruker Daltonics; Bremen, Germany).

### 2.8. Antioxidant Activity

#### 2.8.1. DPPH Assay

The DPPH free radical scavenging activity was determined using a slightly modified Blois method [38]. Methanolic solution of DPPH at concentration 0.3 mM was prepared by dissolving 12.4 mg of DPPH in 100 mL of methanol. The test tubes with DPPH reagent were protected from light by covering with aluminum foil. In a 96-well microplate, 20 μL of each diluted water infusion (1:5, *v*/*v*) or a solution of polyphenol was mixed with 200 μL of DPPH solution. The microplate was incubated at ambient temperature in darkness for 30 min. The absorbance was measured at a wavelength of 517 nm. Each sample was tested in triplicate.

The calibration curve was prepared for gallic acid (at concentrations of 0.06–0.6 µM/mL). Results were expressed as the percentage of DPPH scavenging activity for standards and infusions, and as mM of gallic acid equivalents per gram of dry weight (mM GAE/g d.w.) only for infusions. The percentage of DPPH scavenging activity was calculated as follows:

DPPH scavenging activity [%] = (A_1_ − A_0_)/A_0_ × 100%, (1)

where A_1_ is the mean absorbance of the sample and A_0_ is the mean absorbance of the control sample (DPPH + solvent). Additionally, in order to determine the antioxidant activity of selected standards, IC_50_ values were calculated using linear regression analysis.

#### 2.8.2. ABTS Assay

Antioxidant activity against the ABTS free radical was determined according to a method performed by Chen and Kang [39]. To prepare the ABTS reagent, the following procedure was performed: aqueous solutions of 7 mM ABTS reagent and 2.45 mM potassium persulfate solution were prepared. The two solutions were mixed in a 1:1 proportion and were kept in the dark for 16 h. The obtained reagent (stock) was diluted with deionized water to an absorbance of 0.7 (at a wavelength of 734 nm). Next, 200 μL of ABTS reagent was mixed in a microplate with 2 μL of each diluted water infusion (1:5, *v*/*v*) or a solution of polyphenol. The microplate was incubated at room temperature for 15 min and the absorbance was measured at a wavelength of 734 nm. All samples were tested in three repetitions. 

Gallic acid (at concentrations of 0.06–0.6 µM/mL) was used to prepare a calibration curve. Results were expressed as the percentage of ABTS scavenging activity (for pure compounds and infusions) and mM of gallic acid equivalents per gram of dry weight (mM GAE/g d.w.) only for infusions. The percentage of ABTS scavenging activity was calculated as follows:

ABTS scavenging activity [%] = (A_1_ − A_0_)/A_0_ × 100%, (2)

where A_1_ is the mean absorbance of the sample and A_0_ is the mean absorbance of the control sample (ABTS + solvent). In addition, with the aim of defining the antioxidant activity of selected standards, IC_50_ values were calculated using linear regression analysis.

#### 2.8.3. FRAP Assay

Antioxidant activity using the FRAP assay was measured according to the Benzie and Strain method [40], with slight modifications. The FRAP reagent (stock) was prepared by dissolving 10 mM TPTZ in 40 mM HCl, 20 mM FeCl_3_ and 300 mM acetate buffer (pH 3.6) at the ratio 1:1:10 (*v*/*v*/*v*). Next, 20 μL of each diluted water infusion (1:10, *v*/*v*) or a solution of polyphenol and 200 µL of FRAP reagent were applied to a microplate in triplicate. After 4 min of incubation in darkness at ambient temperature, the absorbance at a wavelength of 593 nm was measured.

The calibration curve was prepared both for gallic acid (at concentrations of 0.1–2 µM/mL) and ferrous ions Fe(II) at concentrations of 0.01–0.6 µM/mL. Results were expressed as mM of Fe(II) per gram of dry weight (mM GAE/g d.w.), and mM of gallic acid equivalents per gram of dry weight (mM GAE/g d.w.) for infusions. For reference compounds, results were calculated as µM of Fe(II) and µM of GAE. 

### 2.9. Antiglycation Activity in BSA-MGO Model

A slightly modified method proposed by Liu et al. [41] was applied to investigate the antiglycation activity. For this purpose, 21.2 μM BSA was incubated with 0.5 mM methylglyoxal and 1.5 mM test compounds in 100 mM PBS, pH 7.4 with 0.02% sodium azide. The reaction mixture was allowed to stand at 37 °C, shaking at 50 rpm, for seven days in closed vials out of the light. Fluorescence intensity of total AGEs after incubation was monitored using a Synergy HTX Multi-Mode Microplate Reader (BioTek Instruments Inc., Winooski, VT, USA) at 360 nm for excitation and 460 nm for emission. Data were acquired using Gen5 Software (BioTek Instruments Inc., Winooski, VT, USA). The measurements from three experiments were taken in triplicate, and the percentage of AGE inhibition was then calculated using the following equation:

Inhibition of AGEs = [1 − (FI_1_/FI_0_)] × 100 [%], (3)

where FI_0_ is the mean fluorescence intensity of the blank sample and FI1 is the mean fluorescence intensity of the sample. Data were analyzed using the Shapiro–Wilk test to assess normality of distribution, followed by one-way analysis of variance (ANOVA) with Tukey’s multiple comparison test using the GraphPad Prism 9 software; *p* values equal to or less than 0.05 were considered significant.

### 2.10. Methylglyoxal Trapping Assay

To investigate the activity of methylglyoxal trapping, the method of Sang et al. [42] was used with a slight modification. The assay consisted of incubating 0.6 mM methylglyoxal with 0.2 mM of the test compound, or *C. incanus* infusion in 100 mM PBS at pH 7.4 and 37 °C, and shaking at 50 rpm to adjust the physiological conditions. The incubation reaction was terminated by adding 2.5 μL of acetic acid (≥99%) and placing the collected samples on ice. Samples were then filtered through Durapore 0.22 μm filters (Millipore) and analyzed using UHPLC-ESI-qTOF-MS to assess the potential of selected compounds to form adducts with MGO. Trapping agent solutions were newly prepared prior to the start of each set of experiments, and the pH of the sodium phosphate buffer was determined immediately before use. Analysis of the formed adducts was carried out according to the method described in Section 2.7. 

### 2.11. Multivariate Modeling

The values of TPC, TFC and DPPH, ABTS, and FRAP antioxidant activity determined in the course of the study were used to create a matrix of parameters (102 × 8). Principal component analysis (PCA) and partial least squares discriminant analysis (PLS-DA) were performed applying PLS-Toolbox (ver. 7.0.3, Eigenvector Research, Wenatchee, WA, USA) in the MATLAB environment (ver. R2010a, MathWorks, Natwick, MA, USA) [43,44]. For modeling purposes the data were autoscaled. The constructed models were cross-validated, applying the leave-one-out (LOO) procedure. To evaluate the quality of the PLS-DA model, the sensitivity and specificity were determined. The diagnostic ability of the obtained classifier was shown in the form of the receiver operating characteristic (ROC) curve.

## 3. Results and Discussion

In the present study, we analyzed 51 commercially available rock rose products, labeled as dietary supplements. Each item consisted of dried *C. incanus* leaves or herb and came from European crops. Among the products, there were 22 with raw plant material from Turkey, 10 from Albania, two from Greece, and 17 had no country of rock rose origin given. Water infusion (otherwise known as hot water extraction) is the most common way of preparing plant material, so we analyzed *C. incanus* infusions. Each product was brewed in duplicate. Because we did not homogenize the plant material, each infusion was considered an individual sample (*n* = 51 × 2). The resulting *C. incanus* infusions were used to investigate how the chemical composition of a given sample affects its antioxidant properties in three in vitro assays. The profiling of rock rose water infusions was conducted with UHPLC-ESI-qTOF-MS. In our study, spectrophotometric tests were conducted to determine total phenolics (TPC), flavonoids (TFC), non-flavonoid polyphenols (TPC-TFC; the difference between TPC and TFC values), and antioxidant activity. The antioxidative power was proven via three assays based on the SET (single electron transfer) mechanism: DPPH, ABTS, and FRAP, commonly used in this type of experiment [45]. Moreover, the content of polyphenols, antiradicals, and reducing effect was considered due to the origin of the plant material. Due to *C. incanus* being a good source of flavonoids known as antiglycation agents [46], we also investigated whether the rock rose components could be able to neutralize the harmful effects of methylglyoxal on bovine serum albumin. Furthermore, we tested the possibility of forming MGO adducts with selected flavonols.

### 3.1. Quantification of Phenolics, Flavonoids, and Non-Flavonoid Polyphenols

The water infusions prepared from *C. incanus* leaves are considered as a source of polyphenols, including flavonoids, proanthocyanidins, ellagitannins, and phenolic acid [18,27]. The total phenolic content was calculated as an equivalent of gallic acid (GAE) because galloyl residues are components of tannins. A similar arrangement of phenolic groups also occurs in the B ring of myricetin derivatives. In the current study, the mean TPC of all the examined infusions was 54.7 (range 35.4–73.1) mg GAE/g d.w. The TPC values were at similar levels in samples from Albania (55.3 mg GAE/g d.w.), Greece (53.6 mg GAE/g d.w.), and Turkey (52.9 mg GAE/g d.w.). However, the data obtained for Greek plant materials may not be precise, due to the limited number of samples. Detailed values, including mean, median, minimum, and maximum of samples with different countries of origin, are presented in Table 1. Viapiana et al. [27] reported significant differences between examined *Cistus* products. The value of TPC measured according to the Folin-Ciocalteu method described in that study was in the range 125–271.7 mg GAE/g d.w. for water extract and 4.1–16.7 mg GAE/g d.w. for herbal supplements. Kuchta et al. [24] reported that the TPC for the sample collected in the Mediterranean region of Turkey was 98.5 mg GAE/g d.w. after first brewing, and decreased to 78.5 mg GAE/g d.w. after the third brewing. The variation may be due to a different manner of extract preparation or the various sites of origin and time of harvest. It was previously observed that the chemical composition of rock rose infusions are affected by different brewing temperature and time [20], different particle size, and the amounts of individual plant parts (leaves, stems) in the dried raw plant material [47]. A larger amount of polyphenols was recorded in dried methanol and water-methanol extracts, 269.3–347.3 mg/g of dry extract weight (prepared on a magnetic stirrer or a Soxhlet apparatus) [48], while in the water-ethanol extracts, the content of polyphenols calculated per gram of dry plant material was comparable or slightly lower, ranging from 36.3 mg GAE/g d.w. for extracts prepared in 30% ethanol for 5 min, to 89.4 mg GAE/g d.w. for 80 min [49]. In a subsequent study, the GAE content of 50% water-ethanol solutions between 5 and 80 min ranged from 41.7 to 98.7 mg GAE per gram d.w. [1]. In all the cited studies, TPC values were evaluated by the spectrophotometric method.

In contrast to Viapian et al. [27], our study showed that only a non-significantly lower phenolic content was observed for plant material from Turkey. However, both studies confirmed that Turkish samples tend to be richer in flavonoids. In our case, the mean TFC value for Turkish samples was 36 mg ME/g d.w. The Albanian and Greek plant materials had TFC at the levels of 28.9 and 24.5 mg ME/g d.w., respectively. The mean content of flavonoids expressed as myricetin equivalents for the overall tested samples was on average 31.7 mg/g d.w., and ranged between 16.7 and 58.7 mg/g d.w. The TFC values calculated per selected flavonol, e.g., quercetin equivalents (QE mg/g d.m.), were determined previously in *C. incanus* water infusions [24,27], as well as in methanol and water-methanol extracts [48] or as rutin equivalents in water-ethanol extracts [49]. The TFC expressed as QE was significantly lower than the results expressed as ME, and was 2.5 mg/g d.w. [24] or within the range of 2.7–4.3 mg/g [27]. Higher flavonoid content was estimated for ethanol extracts of *C. creticus* collected in Morocco, where the TFC values were 53 mg QE/g d.w. [31]. 

Examined samples with the highest phenolic content did not correspond with samples with the highest flavonoid content. In the Turkish plant material, a particularly high concentration of flavonoids was linked with a small amount of non-flavonoid polyphenols (mean 17 mg/g d.w.) and a relatively low TPC:TFC (total phenolics to total flavonoids) ratio. Values above 2 indicated the predominance of non-flavonoids among other polyphenols, and the approximate amount of non-flavonoid polyphenols was derived from the difference between TPC and TFC (Table 1). The TPC:TFC ratio for all analyzed products, regardless of origin, ranged from 1 to 3.1. The mean for Greek raw material was 2.2, Albanian 2, and Turkish 1.5. The difference may be due to the relatively high concentration of tannins in samples including high TPC but low TFC. The presence of compounds classified into the group of ellagitannins (for instance punicalin, punicalagin, terflavin A, and cistusin) in *C. incanus* infusions had been reported in some previous publications [18,23] and is confirmed in Section 3.2. 

### 3.2. UHPLC-ESI-qTOF-MS Profiling of C. incanus Water Infusions

*C. incanus* is a commonly used health-promoting plant and is therefore also relatively well-studied regarding its chemical composition [24]. However, many factors such as growing, storage, and extraction conditions affect what compounds can be found in the prepared water infusion [49]. The biological activity depends mainly on the chemical composition; so, to understand the properties of given plant material, it is first necessary to review the compounds present in it. Among analyzed commercial *C. incanus* products, we selected two based on spectrophotometric studies (Table S1). The first product, Ci9, was an example of plant material with a high content of non-flavonoid polyphenols, and the second, Ci26, was a representative of products most rich in flavonoids. 

The composition of Ci9 and Ci26 water infusions was analyzed using UHPLC-ESI-qTOF-MS to identify phenolic components for further in vitro studies. Chromatography coupled with mass spectrometry showed that the only differences between the two analyzed products concern the concentration of selected compounds and not the qualitative composition. A total of 20 polyphenols were identified based on the available literature (Table 2).

UHPLC-ESI-qTOF-MS revealed that the principal flavonoids present in the rock rose infusion were hexosides and pentosides of flavonols: myricetin and quercetin. Hyperoside (quercetin-3-*O*-galactoside) and myricitrin (myricetin-3-*O*-rhamnoside) were predominant. At lower levels, analogous kaempferol derivatives and tiliroside were also identified in the infusion. Apart from flavonols, the presence of ellagic acid and ellagitannins—punicalin, punicalagin, terflavin A and cistusin—was also detected. The results obtained support previous reports on the phytochemical composition of *C. incanus* leaves [18,20,23,50,51,52,53,54].

### 3.3. Antioxidant Potential In Vitro

#### 3.3.1. Antioxidant Effect of *C. incanus* Water Infusions

DPPH, ABTS, and FRAP are frequently used spectrophotometric methods to measure antioxidant action. Although each method is based on the SET mechanism, the principle of action is different. The DPPH and ABTS assays are based on the reaction of antioxidants with organic radicals. However, the FRAP assay measures reduction in the ferric ion Fe(III) complex to the intensely blue-colored ferrous Fe(II) complex by antioxidants in an acidic medium [55]. The results, including the country of origin, expressed as equivalents per g d.w. or % of inhibition, are shown in Table 3. The results for individual samples are presented in Table S1. 

In the current study, the ability to inhibit free radicals (expressed as mean % of inhibition) was 28.1 (13.3–42.6) % in the DPPH test and 27.4 (7.3–40.3) % in the ABTS test. Results calculated as GAE were 25 (11.4–43.4) mM GAE/g d.w. and 1.4 (0.5–2.8) mM GAE/g d.w. for DPPH and ABTS, respectively. Plant material from Turkey showed the highest % of inhibition, which was 30.9% and 29.4% for DPPH and ABTS, respectively. Albanian and Greek products showed a similar but slightly lower antiradical effect: 25.9% and 24.1% in DPPH, and 25% and 21.8% in ABTS. 

In the FRAP assay, the results ranged from 69.29 to 314.28 mM Fe(II)/g d.w. and the mean value was 134.21 mM Fe(II)/g d.w. The highest reducing activity was observed for Turkish plant material and was 155.2 mM Fe(II)/g d.w., followed by Albanian and Greek samples, which showed a reducing effect of 115.4 and 107.7 Fe(II)/g d.w. The mean value of Fe(II) equivalents obtained in our study was similar to the ones reported by other authors. In the study conducted by Viapian et al. [27], the results for the FRAP assay were 58 to 148.5 mM Fe(II)/g d.w. for hot water extracts and 46.4 to 169.3 mM Fe(II)/g d.w. regarding water-methanol extracts. Kuchta et al. [24] reported that the reducing power of tested *C. incanus* water infusion was 112.6 mM Fe(II)/g d.w. 

In summary, the mean antioxidant effect in each test was the highest for *C. incanus* from Turkey. The Albanian and Greek plant materials showed lower radical scavenging and reducing power action. Moreover, the samples with defined origin exhibit higher antioxidant activity than samples with unknown origin, but in the ABTS assay, the difference was negligible. The differences in antioxidant action of *C. incanus* leaves from different countries are thought to be affected by genetic and environmental factors [27].

It was also confirmed that volatile components from different *Cistus* species (*C. creticus*, *C. salvifolius*, *C. libanotis*, *C. monspeliensis*, and *C. villosus*) could scavenge ROS, which was confirmed using DPPH, ABTS, and FRAP assays. Among the examined species, *C. linanotis* essential oil revealed the highest antioxidant activity in vitro. The remaining oils showed a similar effect. *Cistus* essential oils also demonstrated the potential to inhibit lipid peroxidation, as observed in the β-carotene bleaching test [56].

#### 3.3.2. Antioxidant Activity of *C. incanus* Polyphenols and Their Respective Metabolites

To extend our findings, we investigated the antioxidant effects of selected polyphenols identified on rock rose infusions, as well as their potential gut metabolites. The results obtained were expressed as IC_50_ (half-maximum inhibitory concentration) and % inhibition in DPPH and FRAP assays, and as Fe(II) and GAE in FRAP. The polyphenols were assigned to three groups: flavonol glycosides (myricitrin and hyperoside), flavonol aglycons (kaempferol, quercetin, myricetin), and gut metabolites of ellagitannins, such as depsides EA and urolithins A-C. Their antioxidant activity levels are shown in Table 4. 

The range of antioxidant activity among the selected polyphenols rises in DPPH in the following order: ellagic acid > myricetin ≥ myricitrin > quercetin ≥ hyperoside > urolithin C > kaempferol > urolithin A. Urolithin B remained inactive. The antioxidant effect of polyphenols in the FRAP assay was in a similar order, except for quercetin, which showed a much stronger reducing ability than EA. In ABTS, the series started with myricetin and EA, followed by myricitrin, quercetin, urolithins A and C, hyperoside, kaempferol, and urolithin B. In both the current and previous studies, flavonols were considered to be significant antioxidants [46,57,58]. It was reported that quercetin via ROS scavenging could protect pancreatic β cells against oxidative stress, alleviate micro- and macrovascular complications of diabetes, and improve the antioxidant status of diabetic patients when orally administered [57]. Each of the conducted tests confirms that kaempferol is a less effective antioxidant than myricetin and quercetin. Our results indicate that myricetin is slightly more active than myricitrin, and quercetin than hyperoside, which suggests that aglycones reveal slightly stronger antioxidant properties than their simple glycosides. The same tendency had been previously reported by other authors. For instance, Hopia & Heinonen [59] found that myricitrin is slightly less active than myricetin, and quercetin is more active than its glycosides via a bulk methyl linoleate oxidized assay. Plumb et al. [60] noted that kaempferol and quercetin glycosides reveal a much lower antioxidant effect than aglycones. The activity was 32–39% lower according to kaempferol glycosides, and even 50–72% in the case of quercetin glycosides. Furthermore, Bednarska & Fecka [46] reported that quercetin, diosmetin, and hesperetin aglycones are more active in FRAP assay than their rutinosides. Also, in the case of glycosides such as myricitrin, the lower activity in vitro may not correspond with their activity in vivo since glycosides are transformed by the gut microbiota into a more active aglycone form [61]. 

Ellagic acid is both a component of *C. incanus* water infusions [27] and a metabolite formed in vivo by degradation of ellagitannins with microbiota in the gut [45,62]. Our results showed that EA was the strongest antioxidant among the tested polyphenols in DPPH, the second in FRAP, and the third according to ABTS. EA is poorly available due to its low solubility in water and organic solvents. Nevertheless, its metabolites such as urolithins could reach the tissues to a greater extent and play a beneficial role [45]. The antioxidant properties of urolithins are unfortunately much weaker concerning the effect displayed by EA and flavonols. Exclusively urolithin C with three phenolic groups (including the *ortho*-di-phenolic function) exerted comparable effects to flavonols in three assays. Urolithin A with two phenolic groups located on opposite sides of the biphenyl exhibited relatively high activity only in ABTS. However, a weak effect or almost no effect was observed for urolithin B with one phenolic group in the structure. Alfei et al. [45] suggest that the antioxidative effect of urolithins is not significant since IC_50_ in the DPPH assay of urolithin A was 23-fold higher than that obtained by EA. The comparison performed by Alfei et al. might be inaccurate because the difference was calculated according to the results obtained by various authors. Our results seem to be more promising, as the antioxidant effect of EA was about 7-fold higher in the case of urolithin A and 2-fold higher with reference to urolithin C. 

### 3.4. Antiglycation Potential of C. incanus Flavonols

The antiglycation activity of selected flavonols that are constituents of *C. incanus* water infusions was investigated. For this purpose, an in vitro model using bovine albumin as a target protein and methylglyoxal (MGO) as a glycation inducer was employed. Myricitrin, hyperoside, myricetin, quercetin, and kaempferol were tested. Ellagic acid and urolithins were not included in this experiment because their antiglycative effect has been previously thoroughly studied [34]. Aminoguanidine and metformin (a biguanide antidiabetic agent) were used as reference compounds. Both compounds are considered the most potent glycation inhibitors [63,64]. Nevertheless, we did not examine infusions of *C. incanus* due to the presence of tannins, which interacted with BSA and interfered with measurements.

**Figure 1:**
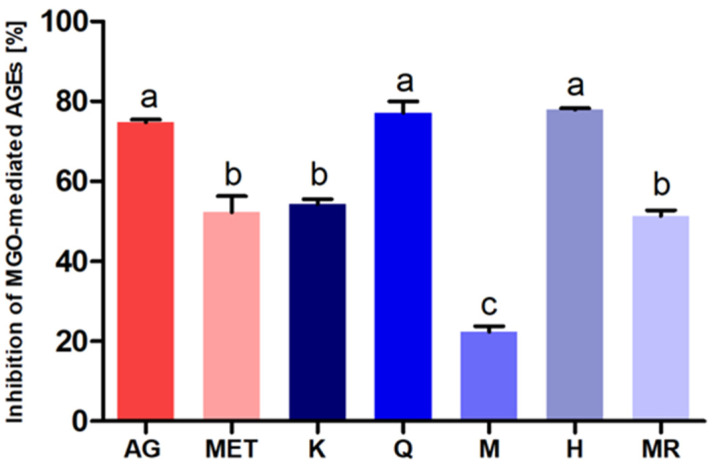
Antiglycation activity after seven days of incubation of BSA with MGO (0.5 mM) and the examined compound (1.5 mM) expressed as % inhibition of MGO-mediated AGE formation. The results are representative of three experiments performed in triplicate ± SD. Values not sharing a common letter are significantly different at *p* < 0.05 by Tukey’s multiple comparisons test. Abbreviations: AG, aminoguanidine; MET, metformin; K, kaempferol; Q, quercetin; M, myricetin; H, hyperoside; MR, myricitrin.

Turning to the results, all investigated flavonols and known inhibitors were observed to have a beneficial effect on the inhibition of advanced glycation end product (AGE) formation in an in vitro model. However, their effectiveness varied. The activity expressed as a percentage of inhibition of AGE formation induced by MGO is shown in Figure 1.

Hyperoside (78 ± 0.8%) and quercetin (76.8 ± 10.8%) exhibited the highest activity in inhibiting the formation of AGEs in vitro. Their antiglycative effect was comparable to the action of aminoguanidine (75.8 ± 1.9%) and significantly higher than that of metformin (49.2 ± 13.3%) used as reference inhibitors. The activities of kaempferol (56.3 ± 3.6%) and myricitrin (51.3 ± 3.5%) reached values close to metformin. Myricetin (23 ± 5.6%) revealed the least ability to inhibit non-enzymatic glycation.

Direct comparison of the investigated compounds’ activities with other authors is challenging because the expression of antiglycation activity is diverse (inhibition of AGE formation %; fluorescence intensity; half maximal inhibitory concentration IC_50_; half-maximal effective concentration EC_50_) [65,66,67]. In addition, different biological models are used to study the antiglycation activity where usually the target protein is BSA, but the glycation factors are varied, e.g., methylglyoxal, glyoxal, glucose, or other reducing sugars [68,69].

In the in vitro model used, the target protein was BSA, and the trigger and accelerator of non-enzymatic glycation was methylglyoxal (0.5 mM), as it is much more reactive than reducing sugars [70]. In an analogous model, Wu et al. [71] found that the inhibitory activity of AGE formation was higher for kaempferol than for quercetin. Perhaps this was related to the high concentration of methylglyoxal used in the assay (100 mM). It is known that quercetin treated with powerful oxidants, such as MGO, can degrade with the formation of quinone forms. So, the activity, which is the result of a specific chemical structure, may decrease. The low antiglycation activity of myricetin could also be due to its instability. The poor stability of myricetin in aqueous solutions was previously reported by Maini et al. [72]. These authors demonstrated that as the number of phenol groups in the B ring increases, the stability of flavonols decreases [72]. Our results were consistent with this observation, as the activity of myricetin was significantly lower than that of quercetin (pyrogallol vs. catechol arrangement in the B ring). Thus, it was found that myricetin was degraded more rapidly, and its antiglycation action was not preserved. Myricitrin, the 3-*O*-rhamnoside of myricetin, showed higher stability and statistically significantly higher activity against AGE formation than its aglycone. Most likely, the presence of the glycone at the C-3 position stabilized the molecule and reduced its susceptibility to rapid degradation. In the case of hyperoside, the difference between its activity and that of the aglycone was not so pronounced. However, its stabilizing effect should also be taken into account. Rohn et al. [73] examined the effect of the sugar moiety on the degradation of quercetin-3-*O*-glycosides, and the results indicated that the combination of quercetin and galactoside (hyperoside) was the most stable compared to rhamnoside (quercitrin) or rutinoside (rutin). 

The inhibitory effect of flavonoids on glycation is thought to be mainly attributable to their antioxidant, metal chelating, and MGO uptake properties [74]. However, in attempts to compare antiglycation activity between compounds, their stability in oxidizing conditions should always be regarded. 

### 3.5. Methylglyoxal Trapping Capacity of C. incanus Flavonols

The methylglyoxal trapping potential was tested under simulated physiological conditions for freshly prepared infusions of *C. incanus* leaves (Ci9 and Ci26) and flavonols representing the components of this plant material (kaempferol, quercetin, myricetin, hyperoside, and myricetin). Urolithins in preliminary tests did not show an MGO capture effect under the conditions used and were therefore not considered for further work. In this experiment, the positive reference substance was quercetin.

The reaction products were analyzed in detail by UHPLC-ESI-qTOF-MS to detect structural modifications of flavonols with MGO uptake properties. That analysis was performed with the Extract Ion Chromatogram (EIC) function, thus scanning for pseudomolecular ions enlarged by 72 Da (mono-adducts) and by 144 Da (di-adducts). MGO-flavonol adducts were observed in negative ion mode. Table 5 contains the results for each flavonol standard and selected *C. incanus* water infusions.

The above experiments showed that both the infusion of *C. incanus* and some of its components possess the ability to capture MGO. In UHPLC-ESI-qTOF-MS, we observed formation of mono-MGO adducts with myricitrin and hyperoside. Among the standards, myricitrin, hyperoside, quercetin, and kaempferol exhibited MGO trapping ability, while no activity was observed for myricetin. The details are as follows.

A 1-h incubation of hyperoside standard with methylglyoxal resulted in five chromatographic peaks. The first was characterized as di-MGO-hyperoside as it was 144 Da larger than that of hyperoside (463 + 144 = 607 Da), corresponding to the attachment of two methylglyoxal moieties. The other four that appeared on the chromatogram later with *m*/*z* at 535 were considered to be isomers of mono-MGO-hyperoside (hyperoside molecular weight increased by 72 Da). Hyperoside has previously shown activity against other RCS, such as phenylacetaldehyde, via an identical mechanism [75].

Myricitrin standard incubation with methylglyoxal for 1 h produced five peaks on the chromatogram with pseudomolecular ions *m*/*z* 535, all of which appeared to be mono-MGO-myricitrin isomers as indicated by their mass increase of 72 Da (463 + 72 = 535 Da). No formation of di-MGO adducts of myricitrin and methylglyoxal was noted. 

In the reaction of kaempferol and methylglyoxal after an hour, the reaction mixture contained four adducts. The first two were identified as isomeric di-MGO-kaempferol based on the mass of the pseudomolecular ion *m*/*z* 429 (285 + 144 = 429 Da). Meanwhile, the other two peaks with a pseudomolecular ion at *m*/*z* 357 were characterized as isomers of monoadducts of kaempferol, with a mass increased by the mass of one MGO molecule (285 + 72 = 357 Da). Our results were in agreement with the previously reported results of Yang et al. [76], which evidenced the ability of kaempferol to form both mono and diadducts with methylglyoxal under simulated physiological conditions.

Quercetin incubated with methylglyoxal resulted in a total of three peaks, two of which were characterized as mono-MGO-quercetin isomers and one as di-MGO-quercetin. Their pseudomolecular ions were at *m*/*z* 373 for the mono-MGO adducts (301 + 72 Da) and 445 (301 + 144 Da) for the di-MGO adduct of quercetin. Quercetin is a compound for which the methylglyoxal-binding activity is well established and documented [77,78].

No methylglyoxal trapping capacity was noted for myricetin. Moreover, after 1 h of incubation, no pseudomolecular ion originating from myricetin was observed, indicating its rapid degradation. Similar observations of the low stability of this compound were confirmed in the study of Maini et al. [72]. Their results showed that myricetin degrades more than 90% in an aqueous solution at room temperature within 3 h. And this phenomenon may explain the absence of the peak corresponding to myricetin in the chromatogram obtained in the current study. Flavonols with three phenol groups in the B ring, such as myricetin, are more vulnerable to rapid decomposition by the *ortho*-quinone formation. 

After 1 h of incubation of Ci26 water infusion with MGO, three product peaks were observed in UHPLC-ESI-qTOF-MS at *m*/*z* 535. MS/MS analysis revealed that the first two peaks between 19 and 20 min were isomers of mono-MGO-hyperoside (463 + 72 = 535 Da), while the third peak was labeled as mono-MGO-myricitrin (463 + 72 = 535 Da) based on comparison with the flavonol standards. For Ci9 infusion, the presence of four peaks at *m*/*z* 535 was noted after the same incubation time. The first to appear was assigned as mono-MGO-hyperoside, based on the retention time of the mono-MGO adduct of the hyperoside standard. In contrast, the other three peaks based on MS/MS analysis were tentatively labeled as isomers of mono-MGO-myricitrin. 

In summary, it can be concluded that flavonol glycosides such as myricitrin and hyperoside were mainly responsible for the anti-MGO activity of *C. incanus* water infusions. No adducts of aglycones, which were present in the rock rose infusions at much lower levels or underwent oxidative changes like the myricetin standard, were observed. Nevertheless, a study by Zhang et al. [79] suggests that under in vivo conditions in mice, myricetin is metabolized to methylated and glucuronosylated derivatives that possess the potential for MGO uptake. As it seems, biotransformation of myricetin to phase II metabolites may further stabilize the molecule.

### 3.6. Correlation between Chemical and Antioxidant Parameters 

Determined contents of phenolics, flavonoids, non-flavonoid polyphenols, and parameters characterizing antioxidant activity of the studied plant material were correlated. Due to the small number of samples, for calculation of Pearson’s correlation coefficient (*R*) the results of Greek plant materials’ analyses were combined together with those of Albanian material. In the studied infusions (*n* = 102), the contents of total phenolic compounds and total flavonoids corelated moderately, and the highest value of the correlation coefficient was reported for Turkish samples (*R* = 0.46). Interestingly, the values of antioxidant activity parameters displayed higher dependency on TFC than on TPC. As data collected in Table 6 show, a strong correlation between the DPPH and FRAP assays with the content of flavonoids was observed; in the case of Albanian and Greek material analysis, *R* reached the values of 0.81 and 0.88, while for Turkish material and the whole sample set the value was 0.7. The correlation coefficients obtained when DPPH, ABTS, and FRAP assays matched with TPC were significantly lower, with values of 0.19–0.25 for all samples, 0.23–0.4 for Turkish, and 0.29–0.54 for Albanian, which suggests that flavonoids are the main group of active compounds responsible for antioxidant activity of *C. incanus* infusions. Supporting this, the content of non-flavonoid polyphenols, expressed here as TPC-TFC, displayed a moderate but negative correlation with all three measures of antioxidant activity. 

For parameters characterizing antioxidant activity of the plant material, the use of different ways of expression can result in high correlations between the measured variables. However, differences in chemical composition of the studied samples incorporate additional variability between variables. Analysis of DPPH and FRAP assays showed high correlations for all samples, i.e., the value of *R* was in the range 0.64–0.8 and 0.63–0.71 for Albanian and Turkish samples. In the case of ABTS and FRAP relationships, a significant decrease in the parameter was observed, with values in the range 0.31–0.45 and 0.28–0.65, respectively (Table S2 in the Supplementary Material). 

Discussing the obtained correlation coefficient values (Table 6 and Table S2 in the Supplementary Material), we can conclude that the antioxidant effect of *C. incanus* water infusions may be related to the occurrence of flavonoids and their reducing power (FRAP), followed by radical scavenging properties (DPPH, ABTS).

### 3.7. Multivariate Classification of C. incanus Samples

The purpose of construction of the PCA and PLS-DA models was to determine whether it is possible to distinguish *Cistus* samples according to their origin, based on a relatively small number of parameters. Additionally, due to the fact that for a significant part of the analyzed samples (*n* = 34) the country of origin was not declared, such models would make it possible to determine their approximate source. Therefore, discrimination models were constructed based only on samples whose origin was known (*n* = 68), in accordance with the manufacturers’ declaration. Due to the small number of data for the Greek material (*n* = 4), no separate class for these samples was created during PLS-DA modeling. 

**Figure 2:**
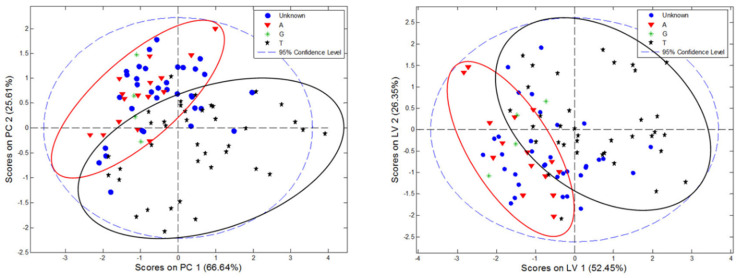
PCA (**left**) and PLS−DA (**right**) score plots for *C. incanus* parameters modeling (A, Albanian; G, Greek, T, Turkish plant material).

Principal component analysis was performed on the matrix of the previously determined parameters, i.e., TPC, TFC, and antioxidant activities, expressed as % of inhibition, without any additional pre-treatment. The first PC described over 66%, while the second one 25.6% of the total variance of the dataset. Distribution of the objects in the PC1/PC2 coordination system, presented in Figure 2, showed a specific grouping of the samples. In general, samples of Turkish origin were characterized by greater variability of the analyzed parameters, which resulted in large dispersion of the objects in the variability space. This can indicate an impact of, e.g., various growing conditions of the leaf, different material treatment, or the impact of its storage on the resulting TPC and TFC contents. Albanian samples created more compact grouping in the scores plot, located practically in one quadrant of the PC1/PC2 coordinate system. However, separation between Turkish and Albanian samples in the scores plot was not complete, suggesting that despite different geographical origins, the expression of polyphenolic compounds was comparable, or the measured antioxidant activity was related to the presence of similar active compounds in the studied plant material. Interestingly, no difference was observed between the location of Greek and Albanian samples in the PC1/PC2 space. On the basis of the constructed PCA model, a set of “unknown” *Cistus* samples was analyzed. Approximately 2/3 of these samples were located in the space of variability characteristic for samples of Albanian origin, and only a few could be classified as purely Turkish.

**Figure 3:**
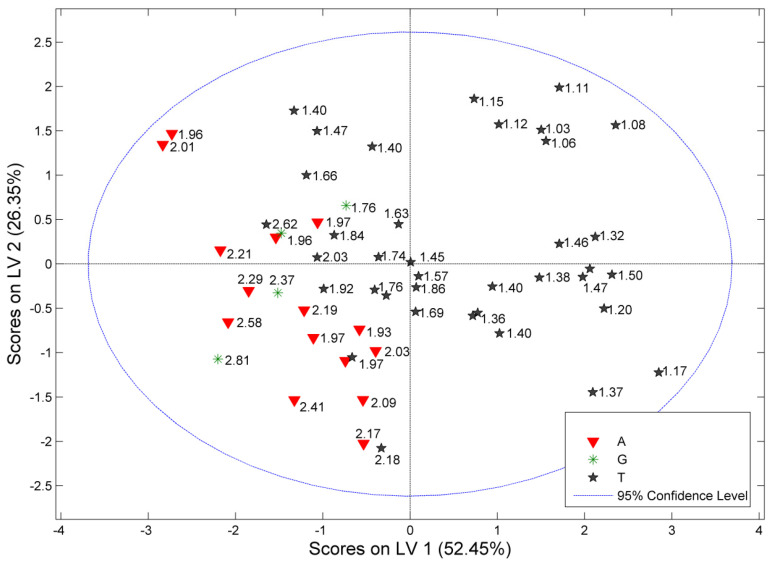
PLS−DA: score plots for *C. incanus* parameters with the values of the TPC:TFC ratio (A, Albanian; G, Greek, T, Turkish plant material).

PLS-DA performed for the parameters matrix resulted in slightly different eigenvalue shares when compared to PCA. The first PLS factor described 52% of the total variance of the dataset, while LV2 and LV3 characterized 26% and 15% of the variability, respectively. The obtained distribution of the objects in the scores plot was similar to that obtained from PCA (Figure 2). Interestingly, an arrangement of samples in the LV1/LV2 coordinate system displayed a strong dependence on the value of the TPC:TFC ratio for the studied samples (Figure 3). The constructed classifier gained the highest separation when three factors were utilized, and was characterized by the sensitivity and specificity in the range 86.5–93.3%. Plots of the errors of classification of the model are shown in Figure S1 in the Supplementary Material, while the receiver operating characteristic (ROC) curves, expressing performance of the classification, are presented in Figure S2 in the Supplementary Material. Quantification of the plant material of an unknown origin based on the constructed model confirmed that a dominant part of them could be assigned to Albanian plant material.

### 3.8. Study Limitations

TPC, TFC, and antioxidant capacity in vitro are parameters difficult to compare with the results obtained by other authors. Samples are prepared differently, depending on the trial. Different types of solvents (water, mixture of water with methanol or ethanol) are used by researchers to prepare extracts. The methods of preparing water infusions also varied. Differences include brewing time and dry to water ratio. Additionally, commercially available herbal supplements are purchased in different countries, and the information about the origin is often omitted by the producer. Furthermore, the procedure for spectrophotometric tests varies across studies and is individually modified by the authors.

## 4. Conclusions

Among the analyzed rock rose samples, Turkish samples were characterized by the highest flavonoid content and the lowest content of non-flavonoid polyphenols, which was associated with higher antioxidant activity in vitro. The compilation of correlation coefficients confirmed that flavonoids are a significant group of compounds responsible for the antioxidant properties of *C. incanus* water infusions. Additional tests using polyphenol standards demonstrated that flavonols and ellagic acid have superior antioxidant potential in contrast to urolithins (intestinal metabolites of ellagitannins).

*C. incanus* flavonols demonstrate the ability to trap methylglyoxal and therefore may be useful in the prevention and early treatment of diseases associated with the overproduction of RCS. Moreover, flavonol glycosides such as myricitrin and hyperoside have the ability to inhibit protein glycation. The effect of hyperoside was comparable to that of the best known antiglycation agent, aminoguanidine, and was even higher than that of metformin. Due to the potent beneficial properties of *C. incanus* and its constituents as antioxidant and antiglycation agents, the application of rock rose infusions in the support therapy of diseases coexisting with oxidative and carbonyl stress could be considered.

The use of chemometric techniques of data treatment for parameters determined on the basis of commonly used spectrophotometric measurements allowed for the discrimination between samples according to their origin. PLS-DA modeling can be used for tentative identification of the place of origin of the *C. incanus* raw plant material with sensitivity and specificity exceeding 86%.

## Figures and Tables

**Table 1:** Total phenolics (TPC), flavonoids (TFC), and non-flavonoid polyphenols (TPC-TF) in *C. incanus* water infusions.

Sample Origin		TPC	TFC	TPC-TFC ^a^ [mg/g d.w.]	TPC:TFC
		GAE [mg/g d.w.]	ME [mg/g d.w.]		
All samples (*n* = 102)	Mean	54.69 ± 8.23	31.73 ± 8.55	23.14 ± 9.73	1.8 ± 0.5
	Median	56.05	30.22	24.4	1.8
	Minimum	35.39	16.7	1.33	1
	Maximum	73.09	58.68	46.18	3.1
Albania (*n* = 20)	Mean	55.3 ± 6.97	28.92 ± 6.32	26.37 ± 6.99	2 ± 0.3
	Median	55.03	27.07	27.97	2
	Minimum	43.24	21.44	12.7	1.3
	Maximum	73.09	41.32	39.44	2.6
Greece (*n* = 4)	Mean	53.57 ± 4.95	24.47 ± 2.19	29.1 ± 7.08	2.2 ± 0.4
	Median	52.72	24.52	28.2	2.2
	Minimum	47.74	21.7	20.6	1.8
	Maximum	61.08	27.14	39.38	2.8
Turkey (*n* = 44)	Mean	52.92 ± 9.13	35.96 ± 9.23	16.96 ± 8.92	1.5 ± 0.4
	Median	53.42	33.98	17.49	1.5
	Minimum	35.39	16.7	1.33	1
	Maximum	69.41	58.68	36.77	3.1

*n*, number of infusions; ^a^, difference between TPC and TFC values.

**Table 2:** UHPLC-ESI-MS data of *C. incanus* infusion components in negative ion mode.

No.	Rt [min]	[M − H]^−^ [*m*/*z*]	MS/MS [*m*/*z*]	Identification	Reference
1	5.52	781.0581		Punicalin *isomers 1* and *2*	[17]
2	5.90	781.0583			[17]
3	13.57	1083.0631/541.0264 ^a^	781, 601, 301	Punicalagin *isomers 1* and *2*	[23]
4	15.90	1083.0637/541.0264 ^a^	781, 601, 451, 301		[23]
5	16.97 17.36	1085.0769/542.026 ^a^	301	Terflavin A	[23]
6	18.08 18.34	1251.0706/625.0285 ^a^	301	Cistusin *isomers 1* and *2*	[23]
7	19.05	300.9841	257, 229	Ellagic acid	[50]
8	19.92	631.098	316/317 ^b^	Myricetin-*O*-galloyl-hexoside (*galactoside* or *glucoside*)	[51]
9	20.44	479.0854	316/317 ^b^	Myricetin-3-*O*-galactoside	[18]
10	20.70	479.0861	316/317 ^b^	Myricetin-3-*O*-glucoside	[18]
11	20.00	493.1377	313, 179	Dimethyl-kaempferol-*O*-hexoside (*galactoside* or *glucoside*)	[52] [50]
12	21.82	449.0751	316/317 ^b^	Myricetin-3-*O*-pentoside (*xyloside* or *arabinoside*)	[53]
13	22.09	463.091	316/317 ^b^	Myricitrin (myricetin-3-*O*-rhamnoside)	[20]
14	22.40	463.0906	300/301 ^b^	Hyperoside (quercetin-3-*O*-galactoside)	[18]
15	22.71	463.091	300/301 ^b^	Isoquercitrin (quercetin-3-*O*-glucoside)	[18]
16	23.74	433.0798	300/301 ^b^	Quercetin-3-*O*-pentoside *isomer 1* (*xyloside* or *arabinoside*)	[53]
17	24.05	433.0801	300/301 ^b^	Quercetin-3-*O*-pentoside *isomer 2* (*xyloside* or *arabinoside*)	[53]
18	24.39	447.0949	300/301 ^b^	Quercitrin(quercetin-3-*O*-rhamnoside)	[20]
19	28.77	609.1293	301	Quercetin-*O*-rhamnoside-*O*-hexoside (*rutin* or other *isomer*)	[20]
20	30.27	593.1348	285	Tiliroside (6″-*O-p*-coumaroylastragalin)	[54]

^a^, [M − 2H]^2−^; ^b^, [Y_0_ − H]^−•^/[Y_0_]^−^ (radical aglycone ion/aglycone ion).

**Table 3:** Antioxidant activity of *C. incanus* water infusions.

Sample Origin		DPPH		ABTS		FRAP	
		Inhibition ^a^ [%]	GAE [mM/g d.w.]	Inhibition ^a^ [%]	GAE [mM/g d.w.]	Fe(II) [mM/g d.w.]	GAE [mM/g d.w.]
All samples (*n* = 102)	Mean	28.12 ± 5.63	24.98 ± 5.68	27.36 ± 5.92	1.39 ± 0.43	134.21 ± 42.72	29.73 ± 9.76
	Median	28.05	24.73	28.33	1.35	128.18	28.33
	Minimum	13.29	11.38	7.31	0.54	69.29	11.39
	Maximum	42.65	43.37	40.31	2.79	314.28	71.89
Albania (*n* = 20)	Mean	25.94 ± 6.16	22.54 ± 5.42	25.01 ± 6.66	1.33 ± 0.47	115.39 ± 28.73	25.12 ± 6.94
	Median	27.1	23.88	26.25	1.29	110.39	24.47
	Minimum	13.29	11.38	7.31	0.54	69.29	11.39
	Maximum	35.45	30.35	36.43	2.58	168.53	37.36
Greece (*n* = 4)	Mean	24.09 ± 1.4	20.63 ± 1.19	21.83 ± 1.68	1.37 ± 0.49	107.72 ± 11	23.80 ± 3.08
	Median	24.13	20.65	22.19	1.11	106.7	23.65
	Minimum	22.09	18.91	19.18	1.05	93.26	19.61
	Maximum	26.03	22.28	23.77	2.21	124.2	28.3
Turkey (*n* = 44)	Mean	30.90 ± 5.31	27.02 ± 4.95	29.41 ± 6.02	1.47 ± 0.45	155.22 ± 47.74	34.53 ± 10.87
	Median	29.56	25.73	30.06	1.43	146.04	32.37
	Minimum	20.35	17.42	17.56	0.84	90.92	20.15
	Maximum	42.65	38.09	40.31	2.79	314.28	71.89

*n*, number of infusions; ^a^, calculated for samples diluted 1:5.

**Table 4:** Antioxidant effect of selected flavonols (aglycones, glycosides), and depsides (ellagitannin metabolites).

Sample	DPPH		ABTS		FRAP	
	IC_50_ [µM]	Inhibition ^a^ [%]	IC_50_ [µM]	Inhibition ^a^ [%]	Fe(II) ^b^ [µM]	GAE ^b^ [µM]
Kaempferol	6.52 ± 0.31	41.17 ± 1.88	8.04 ± 0.14	33.6 ± 0.24	45.99 ± 0.2	6.69 ± 0.06
Quercetin	3.53 ± 0.51	63.06 ± 4.15	4.30 ± 0.66	54.24 ± 8.21	116.59 ± 4.26	24.44 ± 0.73
Myricetin	3.06 ± 0.1	86.13 ± 2.06	3.15 ± 0.18	79.8 ± 6.07	64.67 ± 0.76	12.2 ± 0.22
Hyperoside	3.91 ± 0.2	57.26 ± 2.04	7.97 ± 1.1	32.28 ± 5.13	49.76 ± 1.7	4.84 ± 0.29
Myricitrin	3.31 ± 0.08	78.48 ± 1.96	3.92 ± 0.21	66 ± 2.3	62.72 ± 1.62	11.63 ± 0.48
Ellagic acid	2.35 ± 0.07	91.27 ± 0.13	3.82 ± 0.13	59.66 ± 4.18	100.99 ± 3.69	22.92 ± 1.09
Urolithin A	16.15 ± 0.63	18.11 ± 0.61	5.47 ± 0.56	49.48 ± 6.11	43.13 ± 2.65	5.85 ± 0.78
Urolithin B	<0	<0	27.72 ± 3.54	7.63 ± 2.07	16.41 ± 0.38	<0
Urolithin C	4.77 ± 0.03	54.54 ± 0.43	6.28 ± 0.64	42.3 ± 4.15	59.73 ± 2.04	10.75 ± 0.6

Values averaged from 3 measurements; ^a^, calculates for a final concentration of 5 µM; ^b^, calculated for a final concentration of 9 µM.

**Table 5:** Reaction products of methylglyoxal with flavonols (standard or present in the water infusion) formed after 1 h of incubation in phosphate buffer solution pH 7.4 at 37 °C.

Flavonol or Product	Source	Rt [min]	[M − H]^−^ [*m*/*z*]	Adduct or Precursor
**Kaempferol**	S	32.39	285.0411	
		25.67	429.0818	di-MGO-Kaempferol *1*
		25.77	429.0821	di-MGO-Kaempferol *2*
		27.98	357.0616	mono-MGO-Kaempferol *1*
		28.63	357.0611	mono-MGO-Kaempferol *2*
**Quercetin**	S	28.90	301.0354	
		23.99	445.0788	di-MGO-Quercetin
		25.62	373.0563	mono-MGO-Quercetin *1*
		25.78	373.0564	mono-MGO-Quercetin *2*
**Myricetin**	S	-	-	-
**Hyperoside**	S	22.40	463.0886	
		19.00	607.1288	di-MGO -Hyperoside
		19.65	535.1089	mono-MGO-Hyperoside *1*
		19.88	535.1091	mono-MGO-Hyperoside *2*
		20.48	535.1094	mono-MGO-Hyperoside *3*
		20.70	535.1092	mono-MGO-Hyperoside *4*
**Myricitrin**	S	22.09	463.0902	
		19.91	535.1097	mono-MGO-Myricitrin *1*
		20.24	535.1108	mono-MGO-Myricitrin *2*
		20.31	535.1105	mono-MGO-Myricitrin *3*
		20.48	535.1101	mono-MGO-Myricitrin *4*
		20.59	535.1101	mono-MGO-Myricitrin *5*
**Ci9**	Inf.	22.09	463.0909	Myricitrin
		22.40	463.0906	Hyperoside
		19.67	535.1116	mono-MGO-Hyperoside *1*
		19.90	535.1134	mono-MGO-Myricitrin *1*
		20.24	535.1135	mono-MGO-Myricitrin *2*
		20.31	535.1137	mono-MGO-Myricitrin *3*
**Ci26**	Inf.	22.09	463.0909	Myricitrin
		22.40	463.0906	Hyperoside
		19.65	535.1115	mono-MGO-Hyperoside *1*
		19.88	535.1117	mono-MGO-Hyperoside *2*
		20.24	535.1123	mono-MGO-Myricitrin *2*

S, reference standard; Inf., infusion.

**Table 6:** Correlation between chemical composition and antioxidant effect (as heatmap).

Sample Origin	Method	Unit	TPC	TFC	TPC-TFC ^a^
All (*n* = 102)	TPC	GAE [mg/g]	-		
	TFC	ME [mg/g]	0.28	-	
	TPC-TFC	[mg/g]	0.6	−0.6	-
	DPPH	Inhibition [%]	0.19	0.69	−0.41
	ABTS	Inhibition [%]	0.25	0.49	−0.2
	FRAP	Fe(II) [mM/g]	0.25	0.72	−0.39
Albania + Greece (*n* = 24)	TPC	GAE [mg/g]	-		
	TFC	ME [mg/g]	0.39	-	
	TPC-TFC	[mg/g]	0.61	−0.49	-
	DPPH	Inhibition [%]	0.54	0.81	−0.19
	ABTS	Inhibition [%]	0.48	0.45	0.08
	FRAP	Fe(II) [mM/g]	0.29	0.88	−0.49
Turkey (*n* = 44)	TPC	GAE [mg/g]	-		
	TFC	ME [mg/g]	0.46	-	
	TPC-TFC	[mg/g]	0.54	−0.5	-
	DPPH	Inhibition [%]	0.24	0.51	−0.26
	ABTS	Inhibition [%]	0.23	0.53	−0.27
	FRAP	Fe(II) [mM/g]	0.4	0.71	−0.29

*n*, number of infusions; ^a^, difference between TPC and TFC values.

## Data Availability

Not applicable.

## Supplementary Materials

The following supporting information can be downloaded at: https://www.mdpi.com/article/10.3390/molecules27082432/s1, Figure S1: PLS-DA modeling: plots of average classification errors for *Cistus* samples; Figure S2: PLS-DA modeling: plots of the ROC curves (left panel) and responses (right panel) for classification and cross-validation; Table S1: TPC, TFC and antioxidant activity in vitro of *C. incanus* water infusions; Table S2: Correlation between chemical composition and antioxidant effect (a complete data set).

## Abbreviations

AGEs	advanced glycation end products
BSA	bovine albumin serum
Ci	*Cistus incanus*
EA	ellagic acid
EC_50_	half maximal effective concentration
GA	gallic acid
GAE	gallic acid equivalent
IC_50_	half maximal inhibitory concentration
ME	myricetin equivalent
MGO	methylglyoxal
QE	quercetin equivalent
PCA	principal component analysis
RCS	reactive carbonyl species
PLS-DA	partial least squares-discriminant analysis
ROS	reactive oxygen species
SET	single electron transfer
TFC	total flavonoid content
TPC	total phenolic content
TPC-TFC	total non-flavonoid polyphenols
