# Supplementary material for: Antioxidant and Antiglycation Effects of Cistus × incanus Water Infusion, Its Phenolic Components, and Respective Metabolites
Source: Molecules. 2022 Apr 9;27(8):2432. doi: 10.3390/molecules27082432 (PMC9032239; doi:10.3390/molecules27082432)
Supplement: Supplementary file 1 [file molecules-27-02432-s001.zip › molecules-1658162-supplementary.pdf]

Antioxidant and antiglycation effects of *Cistus* × *incanus* infusions, its phenolic components, and possible metabolites

Karolina Bernacka[1]*, Katarzyna Bednarska[1], Aneta Starzec[1], Sylwester Mazurek[2] and Izabela Fecka[1]

[1] Department of Pharmacognosy and Herbal Medicines, Faculty of Pharmacy, Wroclaw Medical University, ul. Borowska 211, 50-556 Wroclaw, Poland; katarzyna.bednarska@student.umed.wroc.pl (K.Bd.); aneta.starzec@student.umed.wroc.pl (A.S.); izabela.fecka@umw.edu.pl (I.F.)

[2] Laboratory of Chemometrics and Applied Spectroscopy, Department of Chemistry, University of Wrocław, 14 F. Joliot-Curie, 50-383 Wrocław, Poland; sylwester.mazurek@chem.uni.wroc.pl (S.M.)

* Correspondence: karolina.bernacka011@gmail.com (K.Br.);

Supplementary Information

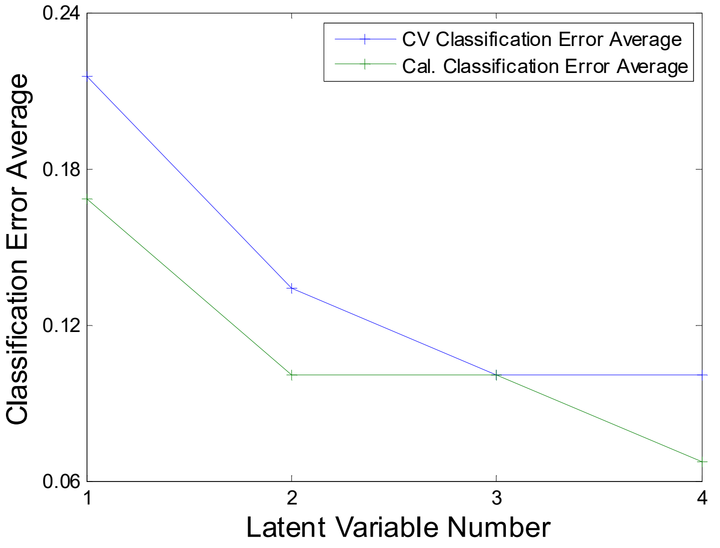

Figure S1. PLS-DA modeling: plots of average classification errors for *Cistus samples*.

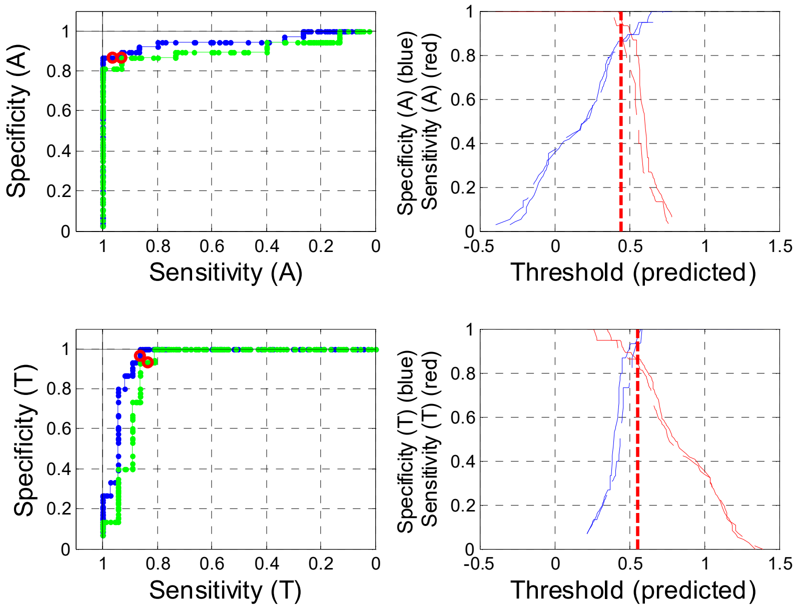

Figure S2. PLS-DA modeling: plots of the ROC curves (left panel) and responses (right panel) for classification and cross-validation; (A) - Albanian and (T)- Turkish samples.

Table S1. TPC, TFC and antyoxidant activity *in vitro* of *C. incanus* (Ci) water infusions.

Product	Sample	Country of origin	TPC	TFC	TPC-TFC	TPC:TFC	DPPH		ABTS		FRAP	
			GAE [mg/g d.w]	ME [mg /g d.w]	-	-	Inhibition[a] [%]	GAE [mM/g d.w]	Inhibition[a] [%]	GAE [mM/g d.w]	Fe (II) [mM/g d.w]	GAE [mM/g d.w]
**Ci1**	1.1	*Albania*	43.24 ± 2.53	21.47 ± 0.45	21.77	2.0	13.29 ± 0.91	11.38 ± 0.78	11.19 ± 2.38	0.54 ± 0.11	69.29 ± 4.34	15.36 ± 0.96
	1.2	*Albania*	44.7 ± 0.69	22.86 ± 0.41	21.84	2.0	13.52 ± 0.48	11.57 ± 0.41	11.72 ± 4.1	0.7 ± 0	82.73 ± 6.44	18.34 ± 1.43
**Ci2**	2.1	*Albania*	49.47 ± 8.92	25.13 ± 1.27	24.34	2.0	19.01 ± 0.48	16.28 ± 0.41	21.43 ± 1.83	1.03 ± 0.09	121.74 ± 2.28	26.99 ± 0.51
	2.2	*Albania*	49.04 ± 4.77	26.82 ± 1.23	22.22	1.8	28.43 ± 1.2	24.34 ± 1.03	28.44 ± 1.68	1.36 ± 0.08	139.82 ± 0.59	30.99 ± 0.13
**Ci3**	3.1	*Albania*	61.33 ± 7.87	41.32 ± 1.2	20.01	1.5	30.65 ± 1.57	26.24 ± 1.34	28.78 ± 6.84	1.38 ± 0.33	147.47 ± 6.73	32.69 ± 1.49
	3.2	*Albania*	57.25 ± 0.86	40.59 ± 0.73	16.66	1.4	35.45 ± 1.43	30.35 ± 1.22	26.12 ± 4.83	1.25 ± 0.23	160.75 ± 4.3	35.63 ± 0.95
**Ci4**	4.1	*Albania*	54.59 ± 6.13	39.92 ± 0.65	14.67	1.4	35.07 ± 1.24	30.03 ± 1.06	32.46 ± 1.84	1.56 ± 0.09	158.27 ± 17.01	34.19 ± 3.08
	4.2	*Albania*	50.02 ± 3.77	37.32 ± 0.07	12.70	1.3	31.83 ± 2.49	27.25 ± 2.13	24.87 ± 2.59	2.39 ± 2.08	168.53 ± 2.69	37.36 ± 0.6
**Ci5**	5.1	*Albania*	53.21 ± 3.8	23.27 ± 1.11	29.94	2.3	24.1 ± 0.12	20.63 ± 0.1	24.11 ± 1.49	1.16 ± 0.07	86.59 ± 1.49	19.19 ± 0.33
	5.2	*Albania*	55.34 ± 2.3	21.44 ± 0.04	33.89	2.6	20.99 ± 1.63	17.97 ± 1.39	25.23 ± 1.52	1.21 ± 0.07	81.78 ± 4.25	18.13 ± 0.94
**Ci6**	6.1	*Albania*	57.47 ± 0.91	29.13 ± 1.25	28.33	2.0	28.51 ± 0.85	24.4 ± 0.72	27.92 ± 3.98	1.34 ± 0.19	94.41 ± 1.82	11.39 ± 16.53
	6.2	*Albania*	57.77 ± 1.9	29.94 ± 0.61	27.83	1.9	29.42 ± 2.16	25.19 ± 1.85	28.12 ± 3.72	1.35 ± 0.18	117.68 ± 11.07	26.08 ± 2.45
**Ci7**	7.1	*Albania*	48.65 ± 1.9	23.34 ± 0.83	25.30	2.1	22.91 ± 0.85	19.61 ± 0.73	23.51 ± 5.22	1.13 ± 0.25	97.31 ± 10.22	23.25 ± 3.32
	7.2	*Albania*	52.6 ± 3.45	23.78 ± 0.23	28.82	2.2	22.05 ± 1.68	18.87 ± 1.44	16.78 ± 1.08	0.8 ± 0.05	89.53 ± 2.49	19.85 ± 0.55
**Ci8**	8.1	*Albania*	61.93 ± 4.8	25.66 ± 2.64	36.27	2.4	25.22 ± 2.28	28.14 ± 11.43	30.8 ± 3.49	2.58 ± 1.92	95.34 ± 2.76	20.75 ± 0.78
	8.2	*Albania*	59.65 ± 3.52	28.76 ± 0.46	30.89	2.1	21.2 ± 1.41	18.15 ± 1.21	29.89 ± 9.41	1.43 ± 0.45	111.67 ± 10.43	24.75 ± 2.31
**Ci9**	9.1	*Albania*	66.43 ± 10.65	31.72 ± 0.94	34.72	2.1	31.83 ± 0.69	27.25 ± 0.59	26.37 ± 4.76	1.26 ± 0.23	130.15 ± 7.52	28.85 ± 1.67
	9.2	*Albania*	73.09 ± 6.78	33.65 ± 0.81	39.44	2.2	31.17 ± 2.13	26.69 ± 1.82	23.09 ± 5.84	1.11 ± 0.28	142.17 ± 5.7	31.51 ± 1.26
**Ci10**	10.1	*Albania*	55.43 ± 3.09	27.32 ± 1.35	28.11	2.0	27.36 ± 1.34	23.42 ± 1.15	36.43 ± 5.64	1.75 ± 0.27	109.12 ± 5.18	24.19 ± 1.15
	10.2	*Albania*	54.73 ± 5.29	25.03 ± 1.1	29.70	2.2	26.84 ± 1.73	22.98 ± 1.48	27.45 ± 1.64	1.32 ± 0.08	103.44 ± 8.69	22.93 ± 1.93
**Ci11**	11.1	*Greece*	50.96 ± 5	26.01 ± 0.29	24.95	2.0	26.03 ± 1.51	22.28 ± 1.29	19.18 ± 0.44	2.21 ± 2.24	106.04 ± 9.4	23.5 ± 2.08
	11.2	*Greece*	47.74 ± 3.67	27.14 ± 0.8	20.60	1.8	24 ± 2.43	20.55 ± 2.08	22.51 ± 5.87	1.08 ± 0.28	124.2 ± 6.77	28.3 ± 1.71
**Ci12**	12.1	*Greece*	61.08 ± 3.67	21.7 ± 1.4	39.38	2.8	22.09 ± 0.36	18.91 ± 0.3	21.88 ± 2.34	1.05 ± 0.11	93.26 ± 5.01	19.61 ± 2.01
	12.2	*Greece*	54.49 ± 2.93	23.03 ± 0.86	31.45	2.4	24.25 ± 2.15	20.76 ± 1.84	23.77 ± 2.55	1.14 ± 0.12	107.37 ± 7.59	23.8 ± 1.68

**Ci13**	13.1	*Turkey*	41.84 ± 3.27	39.37 ± 1.36	2.46	1.1	29.37 ± 0.4	25.15 ± 0.34	29.37 ± 6.1	1.41 ± 0.29	160.46 ± 1.45	33.44 ± 3.68
	13.2	*Turkey*	39.86 ± 3.46	38.53 ± 0.66	1.33	1.0	31.5 ± 0.65	26.96 ± 0.56	30.45 ± 7.32	1.46 ± 0.35	154.95 ± 13.36	34.35 ± 2.96
**Ci14**	14.1	*Turkey*	52.48 ± 6.51	38.54 ± 2.2	13.94	1.4	39.99 ± 1.44	34.23 ± 1.23	37.39 ± 8.61	2.79 ± 1.76	176 ± 13.38	39.01 ± 2.97
	14.2	*Turkey*	50.91 ± 0.67	34.97 ± 3.11	15.94	1.5	37.59 ± 1.52	32.18 ± 1.3	33.36 ± 5.18	1.6 ± 0.25	187.12 ± 24.33	41.48 ± 5.39
**Ci15**	15.1	*Turkey*	37.46 ± 0.67	33.39 ± 1.63	4.06	1.1	26.28 ± 0.69	22.5 ± 0.59	31.74 ± 4.93	1.52 ± 0.24	144.24 ± 12.38	31.97 ± 2.74
	15.2	*Turkey*	35.39 ± 1.29	30.78 ± 2.38	4.61	1.2	25.15 ± 0.62	21.53 ± 0.53	29.69 ± 4.41	1.42 ± 0.21	143.97 ± 5.85	31.91 ± 1.3
**Ci16**	16.1	*Turkey*	36.46 ± 2.14	32.93 ± 4.08	3.52	1.1	28.26 ± 0.75	24.19 ± 0.64	29.84 ± 4.79	1.43 ± 0.23	185.55 ± 7.22	41.13 ± 1.6
	16.2	*Turkey*	41.32 ± 3.71	38.33 ± 1.16	2.99	1.1	24.59 ± 2.33	27.78 ± 11.74	30.78 ± 5.31	1.48 ± 0.25	200.14 ± 17.94	44.36 ± 3.98
**Ci17**	17.1	*Turkey*	40.96 ± 6.58	27.83 ± 0.98	13.13	1.5	25.3 ± 2.9	21.66 ± 2.48	19.23 ± 2.39	0.92 ± 0.11	104.78 ± 5.74	23.22 ± 1.27
	17.2	*Turkey*	41.78 ± 5.46	25.12 ± 1.07	16.66	1.7	26.35 ± 1.34	20.85 ± 3.06	25.15 ± 5.11	1.21 ± 0.24	92.88 ± 4.33	20.59 ± 0.96
**Ci18**	18.1	*Turkey*	38.55 ± 2.71	27.55 ± 1.44	11.00	1.4	32.04 ± 0.99	27.43 ± 0.85	18.55 ± 8.37	0.89 ± 0.4	91.25 ± 2.58	20.23 ± 0.57
	18.2	*Turkey*	39.19 ± 5.02	28.06 ± 0.44	11.13	1.4	35.54 ± 1.58	30.43 ± 1.35	27.26 ± 2.5	1.31 ± 0.12	108.77 ± 1.46	24.37 ± 0.51
**Ci19**	19.1	*Turkey*	50.99 ± 2.39	38.71 ± 0.79	12.28	1.3	38 ± 1.4	32.53 ± 1.2	33.48 ± 2.7	2.67 ± 1.84	192.33 ± 1.67	43.3 ± 1.19
	19.2	*Turkey*	52.78 ± 0.32	38.35 ± 1.43	14.44	1.4	34.76 ± 3.4	28 ± 3.68	35.02 ± 5.3	1.68 ± 0.25	159.25 ± 7.59	35.3 ± 1.68
**Ci20**	20.1	*Turkey*	58.38 ± 1.56	41.59 ± 0.52	16.79	1.4	29.57 ± 0.88	25.32 ± 0.75	25.21 ± 1.21	1.21 ± 0.06	156.57 ± 3.72	34.71 ± 0.82
	20.2	*Turkey*	58.26 ± 1.75	40.22 ± 0.84	18.03	1.5	20.35 ± 2.11	17.42 ± 1.81	17.56 ± 4.93	0.84 ± 0.24	137.97 ± 11.26	30.58 ± 2.5
**Ci21**	21.1	*Turkey*	60.66 ± 3.24	33.71 ± 1.19	26.95	1.8	27.24 ± 1.5	23.32 ± 1.28	18.72 ± 1.66	0.9 ± 0.08	150.61 ± 1.83	33.38 ± 0.41
	21.2	*Turkey*	57.24 ± 4.32	33.94 ± 0.59	23.29	1.7	27.83 ± 3.17	23.83 ± 2.71	28.28 ± 4.25	1.36 ± 0.2	132.58 ± 8.46	29.39 ± 1.88
**Ci22**	22.1	*Turkey*	53.39 ± 6.31	26.25 ± 0.46	27.14	2.0	31.48 ± 1.61	26.95 ± 1.38	21.11 ± 4.26	1.01 ± 0.2	123.56 ± 17.39	27.39 ± 3.85
	22.2	*Turkey*	51.75 ± 3.82	28.11 ± 0.14	23.64	1.8	24.94 ± 2.88	21.35 ± 2.47	20.59 ± 4.48	0.99 ± 0.21	124.93 ± 7.89	27.69 ± 1.75
**Ci23**	23.1	*Turkey*	57.98 ± 5.12	33.01 ± 1.09	24.97	1.8	25.9 ± 0.4	22.17 ± 0.34	21.52 ± 4.32	1.03 ± 0.21	134.33 ± 12.38	29.78 ± 2.74
	23.2	*Turkey*	57.07 ± 6.95	32.89 ± 1.9	24.18	1.7	29.49 ± 2.79	25.24 ± 2.39	17.56 ± 0.32	0.84 ± 0.02	147.84 ± 2.63	32.77 ± 0.58
**Ci24**	24.1	*Turkey*	51.38 ± 2.63	24.72 ± 1.29	26.66	2.1	27.79 ± 0.45	23.79 ± 0.38	30.17 ± 2.27	1.45 ± 0.11	90.92 ± 3.83	20.15 ± 0.85
	24.2	*Turkey*	51.38 ± 2.3	26.7 ± 0.34	24.68	1.9	23.08 ± 2.31	19.76 ± 1.98	29.95 ± 0.68	1.44 ± 0.03	95.5 ± 9.39	21.17 ± 2.08
**Ci25**	25.1	*Turkey*	52.27 ± 2.94	33.38 ± 2.51	18.89	1.6	32.23 ± 0.46	27.6 ± 0.39	30.33 ± 6.8	1.45 ± 0.33	123.18 ± 11.51	27.3 ± 2.55
	25.2	*Turkey*	55.76 ± 4.64	34.01 ± 2.64	21.75	1.6	29.55 ± 0.43	25.3 ± 0.37	33.7 ± 6.04	1.62 ± 0.29	149.67 ± 2.15	33.18 ± 0.48
**Ci26**	26.1	*Turkey*	59.52 ± 3.93	49.69 ± 1.02	9.83	1.2	30.81 ± 1.01	26.38 ± 0.87	31.79 ± 2.13	1.52 ± 0.1	169.62 ± 10.06	37.6 ± 2.23
	26.2	*Turkey*	65.4 ± 5.94	58.68 ± 2.04	6.72	1.1	33.29 ± 1.47	32.75 ± 7.41	35.57 ± 0.73	1.71 ± 0.04	198.6 ± 1.95	44.02 ± 0.43
**Ci27**	27.1	*Turkey*	59.81 ± 3.84	42.72 ± 1.47	17.09	1.4	29.17 ± 2.23	30.4 ± 9.49	31.19 ± 2.81	1.5 ± 0.13	139.82 ± 14.11	30.99 ± 3.13
	27.2	*Turkey*	59.53 ± 10.09	43.78 ± 1.82	15.75	1.4	30.39 ± 1.6	26.02 ± 1.37	27.31 ± 2.97	1.31 ± 0.14	131.39 ± 8.59	29.12 ± 1.9

**Ci28**	28.1	*Turkey*	53.45 ± 3.11	28.79 ± 1.35	24.66	1.9	29.38 ± 1.56	25.15 ± 1.33	30.53 ± 1.29	1.46 ± 0.06	141.87 ± 3.82	31.45 ± 0.85
	28.2	*Turkey*	48.31 ± 1.88	29.59 ± 1.63	18.73	1.6	29.72 ± 2.02	25.44 ± 1.73	27.09 ± 4.57	1.3 ± 0.22	131.44 ± 14.74	28.36 ± 2.67
**Ci29**	29.1	*Turkey*	67.86 ± 1.02	31.1 ± 0.86	36.77	2.2	27.81 ± 1.51	23.81 ± 1.29	33.23 ± 2.94	1.59 ± 0.14	124.5 ± 6.53	27.6 ± 1.45
	29.2	*Turkey*	60.78 ± 4.02	30.79 ± 1.11	29.99	2.0	28.51 ± 1.24	24.41 ± 1.06	27.7 ± 1.14	1.33 ± 0.05	115.58 ± 12.59	25.62 ± 2.79
**Ci30**	30.1	*Turkey*	66.53 ± 2.53	48.64 ± 0.55	17.88	1.4	33.48 ± 0.3	32.85 ± 7.27	34.44 ± 2.18	1.65 ± 0.1	169.36 ± 0.34	39.97 ± 4.2
	30.2	*Turkey*	66.56 ± 4.33	56.83 ± 1.07	9.73	1.2	33.58 ± 0.72	28.75 ± 0.61	33.53 ± 2.11	1.61 ± 0.1	176.68 ± 24.76	39.16 ± 5.49
**Ci31**	31.1	*Turkey*	43.97 ± 4.06	16.8 ± 0.63	27.17	2.6	25.44 ± 2.08	21.78 ± 1.78	28.77 ± 3.9	2.52 ± 1.98	96.1 ± 3.32	21.3 ± 0.74
	31.2	*Turkey*	51.9 ± 3.8	16.7 ± 0.69	35.20	3.1	26.1 ± 1.77	22.34 ± 1.52	25.09 ± 4.98	1.2 ± 0.24	105.25 ± 1.22	23.33 ± 0.27
**Ci32**	32.1	*Turkey*	56.55 ± 2.96	38.36 ± 1.36	18.19	1.5	39.68 ± 0.27	33.97 ± 0.24	29.85 ± 1.96	2.55 ± 1.94	209.74 ± 24.68	49.29 ± 6.2
	32.2	*Turkey*	55.55 ± 1.66	37.09 ± 2.05	18.46	1.5	40.43 ± 0.56	36.82 ± 3.84	33.54 ± 8.18	1.61 ± 0.39	215.08 ± 5.07	47.68 ± 1.12
**Ci33**	33.1	*Turkey*	54.73 ± 0.81	49.82 ± 2.97	4.91	1.1	42.65 ± 0.16	38.09 ± 2.73	39.21 ± 3.38	2.01 ± 1.18	208.79 ± 15.02	46.28 ± 3.33
	33.2	*Turkey*	55.55 ± 2	50.94 ± 1.11	4.61	1.1	42.33 ± 0.28	36.23 ± 0.24	40.31 ± 4.17	1.37 ± 0.14	227.59 ± 5.57	50.45 ± 1.23
**Ci34**	34.1	*Turkey*	67.38 ± 4.11	44.74 ± 1.07	22.64	1.5	35.16 ± 0.27	33.82 ± 6.43	39.69 ± 1.83	1.35 ± 0.06	284.76 ± 20.01	63.12 ± 4.44
	34.2	*Turkey*	69.41 ± 2.96	46.08 ± 2.13	23.34	1.5	37.7 ± 1.37	32.27 ± 1.17	39.19 ± 1.31	1.33 ± 0.04	314.28 ± 30.05	71.89 ± 6.08
**Ci35**	35.1	*unknown*	37.25 ± 0.58	21.72 ± 0.43	15.52	1.7	23.27 ± 1.79	18.62 ± 2.51	19.97 ± 4.23	0.96 ± 0.2	132.71 ± 5.7	29.42 ± 1.26
	35.2	*unknown*	40.07 ± 1.41	22.35 ± 0.67	17.72	1.8	21.57 ± 0.27	18.46 ± 0.23	18.93 ± 5.38	0.91 ± 0.26	94.62 ± 2.18	20.97 ± 0.48
**Ci36**	36.1	*unknown*	42.26 ± 3.1	27.48 ± 0.17	14.78	1.5	25.54 ± 2.16	21.86 ± 1.85	25.2 ± 2.3	1.21 ± 0.11	70.85 ± 4.02	15.71 ± 0.89
	36.2	*unknown*	41.35 ± 5.39	26.03 ± 1.22	15.32	1.6	23.86 ± 1.14	20.43 ± 0.98	28.55 ± 0.96	1.37 ± 0.05	82.82 ± 9.33	18.36 ± 2.07
**Ci37**	37.1	*unknown*	58.99 ± 8.18	29.47 ± 0.97	29.51	2.0	21.92 ± 1.3	18.77 ± 1.11	26.95 ± 2.36	1.29 ± 0.11	76.96 ± 0.41	17.06 ± 0.09
	37.2	*unknown*	57.16 ± 1.05	32.7 ± 1.41	24.46	1.8	25.42 ± 1.46	21.76 ± 1.25	29.34 ± 5.31	1.41 ± 0.25	92.42 ± 1.73	20.49 ± 0.38
**Ci38**	38.1	*unknown*	52.3 ± 0.53	23.54 ± 1.35	28.75	2.2	18.83 ± 2.21	16.12 ± 1.89	22.91 ± 6.47	1.1 ± 0.31	82.94 ± 1.8	18.39 ± 0.4
	38.2	*unknown*	57.16 ± 1.39	22.79 ± 0.46	34.38	2.5	16.46 ± 0.38	14.09 ± 0.33	20.08 ± 0.9	2.24 ± 2.21	81.49 ± 13.95	18.06 ± 3.09
**Ci39**	39.1	*unknown*	51.99 ± 2.41	24.62 ± 1.49	27.37	2.1	25.98 ± 1.51	22.24 ± 1.29	28.37 ± 1.39	1.36 ± 0.07	103.36 ± 9.59	22.91 ± 2.13
	39.2	*unknown*	59.59 ± 9.49	22.32 ± 0.33	37.27	2.7	25.98 ± 1.9	43.37 ± 36.6	30.43 ± 1.14	2.57 ± 1.93	101.26 ± 6.94	22.45 ± 1.54
**Ci40**	40.1	*unknown*	65.67 ± 5.55	23.55 ± 0.53	42.13	2.8	22.44 ± 1.11	41.35 ± 38.34	26.44 ± 2.5	1.27 ± 0.12	107.19 ± 10.23	23.76 ± 2.27
	40.2	*unknown*	56.86 ± 4.5	25.25 ± 0.37	31.61	2.3	23.23 ± 1.94	19.89 ± 1.66	24.24 ± 2.43	1.16 ± 0.12	121.12 ± 9.53	27.96 ± 2.44
**Ci41**	41.1	*unknown*	58.68 ± 2.93	42.91 ± 1.93	15.77	1.4	36.54 ± 1.75	31.28 ± 1.5	30.86 ± 3.66	1.48 ± 0.18	185.39 ± 4.34	41.09 ± 0.96
	41.2	*unknown*	66.59 ± 4.56	44.16 ± 0.29	22.43	1.5	35.8 ± 0.24	30.65 ± 0.21	25.53 ± 2.35	1.22 ± 0.11	184.94 ± 6.85	40.99 ± 1.52
**Ci42**	42.1	*unknown*	58.38 ± 3.08	30.52 ± 0.96	27.86	1.9	29.49 ± 0.54	25.24 ± 0.46	32.48 ± 1.52	1.56 ± 0.07	117.36 ± 9.16	26.01 ± 2.03
	42.2	*unknown*	59.96 ± 9.81	34.33 ± 1.28	25.63	1.8	29.8 ± 0.85	25.51 ± 0.73	29.97 ± 2.6	1.44 ± 0.12	140.51 ± 16.56	31.14 ± 3.67

**Ci43**	43.1	*unknown*	63.97 ± 7.1	37.8 ± 0.86	26.17	1.7	24.07 ± 1.87	20.6 ± 1.6	23.77 ± 4.45	1.14 ± 0.21	114.25 ± 1.72	25.33 ± 0.38
	43.2	*unknown*	64.4 ± 5.9	37.05 ± 3.28	27.35	1.7	29.27 ± 0.8	25.06 ± 0.69	22.06 ± 4.6	1.06 ± 0.22	135.63 ± 8.43	31.15 ± 2.3
**Ci44**	44.1	*unknown*	56.34 ± 1.37	27.28 ± 0.49	29.06	2.1	28.35 ± 0.27	24.27 ± 0.23	28.84 ± 4.79	1.38 ± 0.23	122.15 ± 8.13	27.08 ± 1.8
	44.2	*unknown*	59.5 ± 4.77	29.65 ± 2.39	29.85	2.0	28.34 ± 0.69	24.26 ± 0.59	26.01 ± 3.04	1.25 ± 0.15	117.44 ± 4.7	26.03 ± 1.04
**Ci45**	45.1	*unknown*	55 ± 4.5	36.31 ± 1.09	18.69	1.5	29.25 ± 0.58	30.44 ± 9.36	22.02 ± 6.32	1.06 ± 0.3	148.08 ± 3.67	32.82 ± 0.81
	45.2	*unknown*	59.72 ± 0.85	35.24 ± 1.02	24.47	1.7	31.2 ± 0.91	26.71 ± 0.78	29.89 ± 0.87	1.43 ± 0.04	137.7 ± 8.46	30.52 ± 1.88
**Ci46**	46.1	*unknown*	63.12 ± 1.55	30.49 ± 0.54	32.63	2.1	30.09 ± 0.94	25.76 ± 0.81	33.17 ± 7.44	1.59 ± 0.36	121.47 ± 11.61	26.93 ± 2.57
	46.2	*unknown*	63.7 ± 11.42	30.72 ± 0.62	32.97	2.1	30.11 ± 0.16	25.77 ± 0.13	32.58 ± 0.7	1.56 ± 0.03	128.8 ± 7.13	28.55 ± 1.58
**Ci47**	47.1	*unknown*	56.83 ± 3.2	13.81 ± 14.13	43.01	4.1	23.42 ± 1.46	20.05 ± 1.25	27.88 ± 4.72	1.34 ± 0.23	88.37 ± 0.72	19.59 ± 0.16
	47.2	*unknown*	63.27 ± 7.81	23.2 ± 1.43	40.08	2.7	24.72 ± 0.82	21.16 ± 0.7	28.84 ± 5.07	1.38 ± 0.24	102.79 ± 2.38	22.79 ± 0.53
**Ci48**	48.1	*unknown*	58.29 ± 2.34	36 ± 1.08	22.28	1.6	27.29 ± 1.04	23.36 ± 0.89	31.08 ± 1.66	1.49 ± 0.08	144.96 ± 1.48	32.13 ± 0.33
	48.2	*unknown*	58.59 ± 5.62	38.93 ± 1.35	19.66	1.5	29.94 ± 0.57	25.63 ± 0.48	30.48 ± 4.12	1.46 ± 0.2	140.85 ± 5.1	31.22 ± 1.13
**Ci49**	49.1	*unknown*	57.37 ± 1.38	22.89 ± 0.6	34.49	2.5	22.66 ± 1.04	19.4 ± 0.89	21.41 ± 3.13	1.03 ± 0.15	101.48 ± 8.85	22.49 ± 1.96
	49.2	*unknown*	56.52 ± 4.26	23.23 ± 1.35	33.29	2.4	23.2 ± 0.49	19.86 ± 0.42	16.44 ± 1.77	0.79 ± 0.09	104.11 ± 3.22	23.08 ± 0.71
**Ci50**	50.1	*unknown*	49.86 ± 3.43	25.41 ± 0.79	24.46	2.0	26.92 ± 0.5	29.11 ± 10.51	22.3 ± 5.96	1.07 ± 0.29	127.56 ± 5.98	28.28 ± 1.33
	50.2	*unknown*	49.96 ± 3.36	25.02 ± 0.73	24.93	2.0	29.63 ± 1.57	25.36 ± 1.34	28.48 ± 1.73	1.37 ± 0.08	132.4 ± 6.66	29.35 ± 1.48
**Ci51**	51.1	*unknown*	60.6 ± 2.02	20.64 ± 0.33	39.95	2.9	22.16 ± 0.54	26.39 ± 12.86	32.19 ± 2.63	1.09 ± 0.09	184.96 ± 16.52	41 ± 3.66
	51.2	*unknown*	68.47 ± 2.4	22.29 ± 0.91	46.18	3.1	26.88 ± 0.95	23.01 ± 0.81	31.26 ± 6.47	1.06 ± 0.22	192.18 ± 6.29	42.6 ± 1.39

Note: a, calculated for samples diluted 1:5

Table S2. Correlation between chemical composition and antioxidant effect (a complete data set).

Sample origin		TPC	TFC	TPC-TFC	DPPH Inhib.	DPPH GAE	ABTS Inhib.	ABTS GAE	FRAP Inhib.	FRAP GAE
	**TPC**	-								
	**TFC**	0.28	-							
	**TPC-TFC**	**0.60**	**-0.60**	-						
	**DPPH** Inhib.	0.19	**0.69**	-0.41	-					
All	**DPPH** GAE	0.27	0.55	-0.23	**0.80**	-				
(*n* = 102)	**ABTS** Inhib.	0.25	0.49	-0.20	**0.65**	**0.62**	-			
	**ABTS** GAE	0.06	0.21	-0.13	0.42	0.47	0.57	-		
	**FRAP** Inhib.	0.25	**0.72**	-0.39	**0.71**	**0.62**	0.58	0.23	-	
	**FRAP** GAE	0.24	**0.71**	-0.38	**0.69**	**0.61**	0.56	0.23	**0.99**	-
	**TPC**	-								
	**TFC**	0.39	-							
	**TPC-TFC**	**0.61**	-0.49	-						
Albania	**DPPH** Inhib.	0.54	**0.81**	-0.19	-					
+Greece	**DPPH** GAE	0.58	**0.76**	-0.11	**0.97**	-				
(*n* = 24)	**ABTS** Inhib.	0.48	0.45	0.08	**0.68**	0.72	-			
	**ABTS** GAE	0.21	0.33	-0.08	0.48	0.61	0.55	-		
	**FRAP** Inhib.	0.29	**0.88**	-0.49	**0.80**	0.74	0.45	0.36	-	
	**FRAP** GAE	0.22	**0.79**	-0.47	**0.69**	**0.64**	0.37	0.31	**0.95**	-
	**TPC**	-								
	**TFC**	0.46	-							
	**TPC-TFC**	0.54	-0.50	-						
	**DPPH** Inhib.	0.24	0.51	-0.26	-					
Turkey	**DPPH** GAE	0.28	**0.61**	-0.31	**0.95**	-				
(*n* = 44)	**ABTS** Inhib.	0.23	0.53	-0.27	**0.63**	**0.67**	-			
	**ABTS** GAE	0.00	0.17	-0.16	0.50	0.48	**0.61**	-		
	**FRAP** Inhib.	0.40	**0.71**	-0.29	0.63	**0.71**	**0.65**	0.27	-	
	**FRAP** GAE	0.41	**0.70**	-0.27	0.63	**0.71**	**0.64**	0.28	**1.00**	-
